# Cysteine, methionine, and pantothenic acid remodel the *Saccharomyces cerevisiae* transcriptome and volatile sulfur compound metabolome during alcoholic fermentation

**DOI:** 10.1093/femsyr/foag022

**Published:** 2026-07-07

**Authors:** Rafael Jiménez-Lorenzo, James D Duncan, Valérie Nolleau, Vincent Farines, Jean-Marie Sablayrolles, Audrey Bloem, Carole Camarasa

**Affiliations:** UMR SPO, Université de Montpellier, INRAE, Institut Agro, 34060 Montpellier, France; UMR SPO, Université de Montpellier, INRAE, Institut Agro, 34060 Montpellier, France; UMR SPO, Université de Montpellier, INRAE, Institut Agro, 34060 Montpellier, France; UMR SPO, Université de Montpellier, INRAE, Institut Agro, 34060 Montpellier, France; UMR SPO, Université de Montpellier, INRAE, Institut Agro, 34060 Montpellier, France; UMR SPO, Université de Montpellier, INRAE, Institut Agro, 34060 Montpellier, France; UMR SPO, Université de Montpellier, INRAE, Institut Agro, 34060 Montpellier, France

**Keywords:** *Saccharomyces cerevisiae*, volatile sulfur compound biosynthesis, methionine, cysteine, pantothenic acid deficiency, wine, alcoholic fermentation

## Abstract

Yeast nitrogen and vitamin nutrition are fundamental levers for managing wine quality, yet the specific mechanisms linking nutrient availability to sulfur aroma formation remain poorly understood. This study explored the impact of methionine, cysteine, and pantothenic acid (vitamin B5)—nutrients with direct, pivotal roles in sulfur metabolic pathways—on the *Saccharomyces cerevisiae* transcriptome and VSC metabolome during fermentation. Our findings reveal that methionine and cysteine catabolic routes act as isolated compartments; the genes responsible for bridging these two pathways remain transcriptionally silent in the presence of high cysteine or methionine availability. This lack of metabolic crossover leads to highly specific VSC signatures. Methionine exerts only a limited influence on global gene expression and primarily drives the production of methylthio-compounds via the Ehrlich pathway. Cysteine triggers a starvation-like transcriptomic response and promotes a diverse range of thiols and thioesters. Pantothenic acid deficiency compromised yeast growth and fermentation efficiency, triggered extensive transcriptional changes in sulfur assimilation pathways, effectively redirecting flux toward non-Ehrlich catabolic products. Overall, this study provides a robust mechanistic basis for how sulfur amino acid and pantothenic acid levels can be targeted to modulate wine aroma profiles and prevent the development of reductive off-flavors.

## Teaser

Sulfur amino acids as sole nitrogen sources and pantothenic acid deficiency induce distinct transcriptional and metabolic changes in *Saccharomyces cerevisiae*, affecting sulfur metabolism, nitrogen management as sulfur aroma compounds production during alcoholic fermentation.

## Introduction

Sulfur is an essential nutrient for the biochemical functioning of living organisms, comparable to carbon, nitrogen, and oxygen. Within proteins, sulfur is incorporated via cysteine and methionine, stabilizing tertiary and quaternary structures through disulfide bonds. It is also a core component of iron–sulfur clusters involved in DNA repair, biosynthesis, and central carbon metabolism, including the tricarboxylic acid (TCA) cycle. Additionally, sulfur supports the biosynthesis of vitamins biotin (B7) and thiamine (B1), cofactors required for fatty acid, carbohydrate, and amino acid metabolism as well as for energy production and oxidative stress protection, respectively (Perli et al. 2020, Labuschagne and Divol 2021).

To support growth, *Saccharomyces cerevisiae* can assimilate sulfur from inorganic sources (sulfate, sulfite) via the sulfate reduction sequence (SRS) and from organic sources including cysteine and methionine. The SRS is the primary route of sulfide production, reducing sulfites to sulfide in an NADPH-dependent reaction. The sulfide then condenses to form the key precursor homocysteine, which is vital for synthesizing all sulfur-containing amino acids, cysteine and methionine. However, an imbalance between sulfide formation and its subsequent condensation with O-acetylhomoserine (OAH) to form homocysteine can lead to intracellular sulfide accumulation (Jiranek et al. 1995). Crucially, the SRS is a tightly regulated, multi-functional pathway; its activity is finely modulated by intracellular cysteine levels, via transcription factors including Met4p and Met28p (Thomas et al. 1992, Kuras et al. 1996, Blaiseau et al. 1997, Blaiseau and Thomas 1998, Hansen and Johannesen 2000) and overall amino acid availability (Gcn4p-mediated regulation [Natarajan et al. 2001]), with imbalance potentially leading to sulfide accumulation (Jiranek et al. 1995).

Sulfur metabolism in yeast also underpins the formation of volatile sulfur compounds (VSCs), which have a major impact on the organoleptic properties of fermented beverages, including wine. VSCs include thiols, thioesters, sulfides, disulfides, and methyl- or ethylthio derivatives. Their formation results from the catabolism of methionine, cysteine, and homocysteine, and reflects an extension of the same regulatory and metabolic networks that govern sulfur assimilation and amino acid synthesis (Moreira et al. 2010, Franco-Luesma and Ferreira 2014). These amino acids and their precursor, homocysteine, may be channelled into the Ehrlich pathway as substrates, leading to the formation methionol, 3-methylthiopropionic acid, 3-methylthiopropyl acetate (3MTPAc), 2-mercaptoethanol (2ME), and 3-mercaptopropanol (3MP) (Perpete et al. 2006, Vermeulen et al. 2006, Landaud et al. 2008). Furthermore, a methionine-induced demethiolase activity, cleaving methionine to methanethiol, has also been identified in yeast (Arfi et al. 2002, Perpete et al. 2006), although the responsible gene for this activity has not yet been identified. Cysteine is a key H₂S source, as it is metabolized into pyruvate, ammonium and sulfide via three distinct metabolic pathways mediated by Cys3p/Cys4p, Irc7p, and Tum1p, while acetylation of thiols to thioesters, potentially via Atf1p/Atf2p, adds to VSC diversity (Singh et al. 2009, Hopwood et al. 2014, Santiago and Gardner 2015, Huang et al. 2016). Despite these findings, the molecular and regulatory bases of VSC production remain incompletely characterized.

Given the complexity of the metabolic network involved in the formation of VSCs, many fermentation parameters as well as chemical reactivity, may affect their formation during wine alcoholic fermentation. The most significant factors are yeast assimilable nitrogen (YAN), cysteine, methionine, and pantothenic acid availability, whereas SO₂, sugar, and pH are generally less influential (Jimenez-Lorenzo et al. 2021). For instance, cysteine supplementation increases H₂S and associated catabolites [e.g. 2ME, 2-methylthioethanol (2MTE), ethylthioacetate] and unexpectedly, S-methylthioacetate, whereas methionine supplementation predominantly enhances Ehrlich pathway products. Pantothenic acid deficiency elevates H₂S and methionine-derived metabolites while reducing thioester formation (Edwards and Bohlscheid 2007, Jimenez-Lorenzo et al. 2021).

Despite the recognized impact of sulfur metabolism on yeast physiology and wine aroma, the understanding of the dynamics of sulfur metabolism and the regulation of volatile sulfur metabolite production remains limited. This study aimed to investigate the effects of cysteine or methionine as sole nitrogen sources on the metabolic flux through sulfur metabolic pathways in *S. cerevisiae* under simulated wine-making conditions. Additionally, the impact of pantothenic acid deficiency on VSC production and yeast fermentation kinetics was assessed. Comparative transcriptomic analysis was conducted to identify key candidate genes involved in VSC biosynthesis. By addressing these aspects, this study aims to contribute to a deeper understanding of the genetic and metabolic determinants of sulfur volatile formation and their relevance in wine aroma.

## Material and methods

### Yeast strain and preculture

The *S. cerevisiae* yeast strain LMD17 (Lallemand Inc., Montreal, QC, Canada) was used in all fermentations. Starter cultures were prepared by inoculating a single colony into 5 ml of Yeast Peptone Dextrose (YPD) medium. Strains were grown overnight at 28°C with agitation at 190 rpm. Tubes were centrifuged (4500 rpm, 5 min, 4°C) to obtain a cell pellet which was subsequently washed with 15 ml of NaCl solution (9 g/l) in MilliQ® water. Finally, 1 × 10^6^ cells/ml were inoculated into each fermentation vessel.

### Fermentation media

Fermentations were performed in a synthetic grape must medium containing 210 g/l of sugar in a ratio of 50/50 of glucose and fructose (Table S1). This juice (pH 3.4) mimics typical grape juice conditions and was adapted from Rollero et al. (2015). The conditions of the medium for each treatment differed in the source and quantity of nitrogen (Table S2). To satisfy yeast requirements during anaerobic growth, 2 mg/l of β-phytosterols in a solution of Tween 80 and ethanol (1:1, v/v) were added.

#### Control medium

The control synthetic medium (SM200) contained 200 mg/l of yeast assimilable nitrogen (YAN) in the form of ammonium chloride (Sigma Aldrich, St-Quentin-Fallavier, France) and a mix of amino acids as mentioned in Table S2. No other adjustments were made.

#### Amino acid as a sole nitrogen source media

For treatments with either methionine (MET) or cysteine (CYS) as the sole nitrogen source, the SM200 nitrogen source was replaced with 200 mg N/L provided exclusively by each respective amino acid.

#### Pantothenic acid deficient medium:

For the treatment with pantothenic acid limitation (B5 Def), the SM200 nitrogen source was used, however, pantothenic acid reduced to 10 µg/l, instead of the standard 1 mg/l.

### Transcriptome analysis conditions

For the transcriptome analysis, a higher nitrogen supplementation was necessary to ensure complete fermentation, since single amino acid nitrogen sources resulted in sluggish growth under standard conditions. To investigate the transcriptomic regulation of methionine and cysteine metabolism, and the impact of pantothenic acid deficiency a synthetic must containing 250 mg YAN/l provided exclusively in the form of cysteine or methionine. Pantothenic acid limitation was replicated as above, with the concentration of this vitamin reduced to 10 µg/l rather than 1 mg/l. This increase in YAN (from 200 mg/l in the initial fermentations to 250 mg/l) was specifically adopted to enable robust growth for transcriptome analyses.

### Fermentations conditions for transcriptomic analysis

Transcriptome experiments were conducted using 1 l fermentation vessels equipped with Muller valves. Fermentations were performed in triplicate at 20°C under anaerobic conditions with continuous stirring (300 rpm). The fermentation progress was monitored via CO_2_ release, recorded automatically every 20 min (Sablayrolles et al. 1987). The CO_2_ production rate was calculated using a sliding-window second-order polynomial fit applied to the last 11 measurements in a custom-developed LabView application. Fermentation progress was monitored manually by measuring weight loss, as a proxy for CO_2_ release. Fermentations were considered complete when the CO_2_ production rate was less than 0.01 g/l/h.

### Cell population

Cell counts were determined using an electronic particle counter (Multisizer 3 Coulter Counter, Beckman Coulter) based on changes in electrical resistance caused by the passage of non-conductive particles in suspension in an electrolyte (Isoton, Beckman Coulter) solution as they pass through an aperture (Allen 1990). The samples were diluted to maintain cell concentrations within the linear detection range (20000–80 000 cells/ml) and disrupted by sonication prior to measurement with an ultrasonic generator (Branson Sonifier, model 250).

### Microarray analysis

Gene expression was analysed under the four independent conditions: the SM200 control, the methionine-supplemented synthetic must, the cysteine-supplemented synthetic must and the pantothenate-deficient synthetic must. Samples were taken at two times during fermentation, namely at 5 g and 45 g of CO_2_ release. These times correspond to the growth and stationary phases of fermentation, respectively. A total of 1 × 10^9^ cells were sampled from each fermentation.

Cells were collected by centrifugation (3000 rpm, 2 min, 4°C) in a temperature-controlled centrifuge and washed with diethylpyrocarbonate (DEPC)-treated water to inhibit RNases and immediately frozen in a methanol bath at –80°C. Total RNA was extracted by mechanical lysis using 0.3 mm beads in a solution of Trizol following the protocol described by Chomczynski and Sacchi (1987). RNA was precipitated in a solution of 75% ethanol (v/v) and recovered by centrifugation (10 000 rpm, 5 min, 4°C). The pellets were dried with Speed-Vac, rehydrated in DEPC-treated water and further purified using the RNeasy kit (Qiagen, Hilden, Germany). The RNA concentration was quantified using NanoDrop, and the integrity assessed with the RNA Nano 6000 Assay Kit on the 2100 Bioanalyzer system (Agilent Technologies, Santa Clara, CA, United States).

For the micro-array analysis, 100 ng of purified RNA was converted to cDNA and labelled with the Cy3 fluorochrome using the Low input Quick Amp Labelling One-Color Kit (Agilent Technologies) and purified with the RNeasy Mini Kit (Qiagen, Hilden, Germany). Hybridization was performed with the Gene Expression Hybridization kit (Agilent Technologies) by loading 600 ng of labelled RNA onto single-color Agilent Microarray Chips and incubating overnight in a rotatory oven at 65°C for 17 h. Each slide contained eight hybridization zones, covering 6200 *S. cerevisiae* genes, supplemented by 39 additional genes identified in Lalvin EC1118®.

### Transcriptomic data acquisition and statistical analysis

Hybridization signals were detected using a GenePix 4000B laser scanner (Axon Instruments), and array images were acquired and quantified with integrated GenePix Pro 7 software (Molecular devices). Gene expression differences were evaluated based on the intensity of fluorescence. Statistical analyses were performed in R (version 4.0.4). Data normalization across arrays was carried out using the quantile method in the limma package (Smyth and Speed 2003). Principal component analyses (PCA) were conducted with the FactoMineR package (Lê et al. 2008). The differential analysis was performed using the limma package, applying a linear model to each gene while accounting for duplicates on the slides. Genes were considered significantly differently expressed when |log_2_FC| ≥ 1.0 and *P* < 0.05. In some cases where quadruplicate probes were present with distinct identifiers, they were treated as separate genes. Comparisons of differentially expressed genes (DEGs) across conditions were visualised using Venn diagrams generated with Venny v2.1 (Oliveros 2015; https://bioinfogp.cnb.csic.es/tools/venny/index.html).

For functional enrichment, the list of significant genes was analysed using the web-based tool GeneCodis (http://genecodis.cnb.csic.es/) with an adjusted *P*-value ≤ 0.05 and visualized using volcano plots using VolcaNoseR (Goedhart and Luijsterburg 2020.) The annotated genes were classified by biological processes and metabolic pathways using the Gene Ontology (GO) database and the KEGG database (Kyoto Encyclopedia of Genes and Genomes https://www.genome.jp/kegg/pathway.html). Redundant GO terms were removed using the REVIGO web server (Supek et al. 2011), and the enriched categories were further visualized and examined by condition and fermentation phase using Sankey plots and dot plots generated with the SRplot web-based tool (Tang et al. 2023).

## Results and discussion

We previously demonstrated that variations in methionine and cysteine availability, as well as a deficiency of pantothenic acid, significantly altered the VSC profile produced by *S. cerevisiae* LMD17 during fermentation (Jimenez-Lorenzo et al. 2021). To further explore these distinct production patterns, we investigated the VSC metabolic network and its regulation during fermentation. We compared the transcriptional response of *S. cerevisiae* under four distinct conditions: a control SM200 medium, methionine as a sole nitrogen source (MET), cysteine as sole nitrogen sources (CYS), and growth under limiting pantothenic acid concentrations. Comparative mRNA profiling was conducted at two key time points: the growth phase, indicated by 5 g/l CO_2_ release and coinciding with nitrogen source consumption, and the stationary phase at 45 g/l CO_2_ release, when growth and nitrogen assimilation had ceased.

### Fermentation kinetics

**Figure 1:**
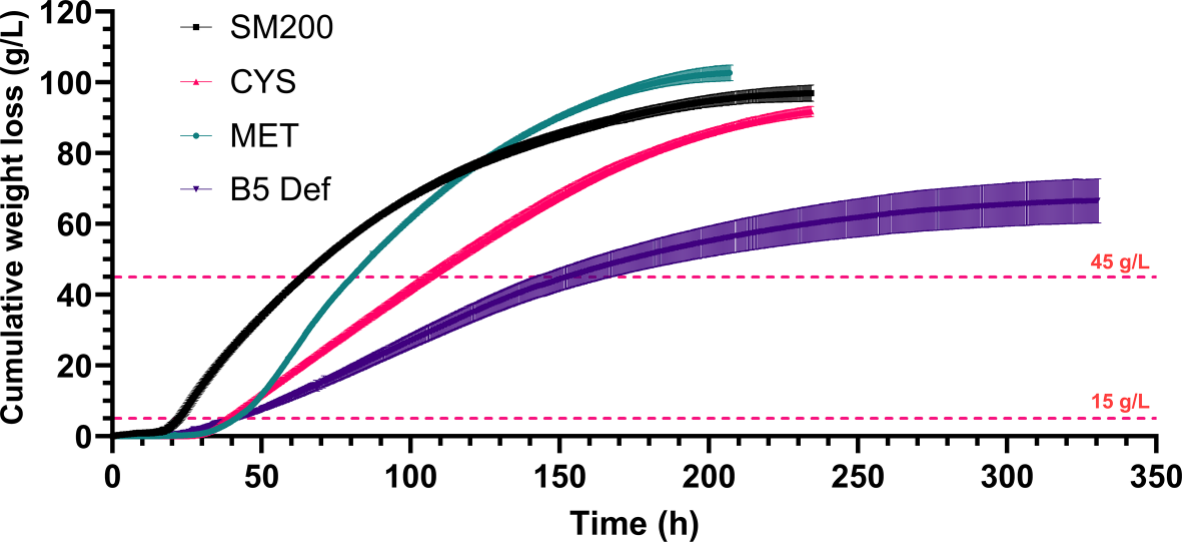
The cumulative weight loss primarily attributed to CO2 release during fermentation of synthetic grape must by *S. cerevisiae* yeast LMD17 in each treatment. Legend: SM200, control medium; CYS, cysteine as sole nitrogen source; MET, methionine as a sole nitrogen source; B5 Def, limiting pantothenic acid (10 μg/l). Samples were taken at 5 g/l and 45 g/l CO2 released (dashed lines) during fermentation. Each data point depicts the mean values of triplicates, while the error bars represent the standard deviation among the triplicate samples.

The cumulative weight loss due to CO_2_ release was monitored throughout fermentation. We observed significant variations in the fermentation kinetics for each treatment until nitrogen source depletion (Fig. 1). Specifically, the MET treatment demonstrated the highest fermentation rate (Table S3), followed by the control SM200 condition, with the control also having the shortest lag phase. The B5 Def treatment consistently resulted in slower fermentation, suggesting a limiting effect. During CYS conditions, a pronounced increase in lag phase duration was observed, accompanied by an approximately two-fold reduction in maximum fermentation rate compared to the control. Nevertheless, similar to the MET condition, we observed a higher stationary phase fermentation activity in the CYS treatment relative to the control (Table S3, R70). This suggests that sulfur amino acid nitrogen sources sustain late-stage fermentation activity despite impairing early fermentation kinetics.

The control condition (SM200) exhibited the shortest lag phase of all treatments, suggesting an advantage conferred by a complete and balanced nutrient environment. Pantothenic acid deficiency had the most marked and broad impact on fermentation performance of all treatments, consistently resulting in sluggish fermentation. We observed a slower fermentation rate, an extended lag phase, and a prolonged overall fermentation duration during B5 deficiency (Table S3). Indeed, this treatment was not able to ferment all sugars, confirming that B5 availability is an important contributor to fermentative efficiency in *S. cerevisiae* (Evers et al. 2023), likely reflecting its essential role as a precursor for Coenzyme A (CoA) biosynthesis and central metabolic function (Leonian and Lilly 1942, Leonardi et al. 2005).

### Production of major metabolites

The primary metabolites were quantified at the end of fermentation for each treatment (Table 1). Broadly, the SM200, MET, and CYS conditions performed similarly by consuming nearly all sugar resulting in similar ethanol yields with no statistically significant differences observed for these parameters. However, the B5 deficiency condition was the clear outlier, consuming significantly less sugar (153 g/l) and producing substantially less ethanol (8.7% v/v), reflecting the sluggish fermentation kinetics described above.

**Table 1:** The primary metabolites were quantified at the end of fermentation.

Parameter (g/l)	SM200	MET	CYS	B5 Def
Consumed sugar	209.62 ± 0.14^a^	209.64 ± 0.03^a^	206.12 ± 0.12^a^	153.05 ± 7.65^b^
Ethanol	101.04 ± 1.2^a^	100.07 ± 1.46^a^	95.44 ± 4.11^a^	68.72 ± 4.8^b^
Glycerol	7.12 ± 0.18^c^	7.07 ± 0.17^c^	8.22 ± 0.24^b^	9.21 ± 0.31^a^
Succinic acid	1.96 ± 0.05^a^	0.18 ± 0.07 c	1.08 ± 0.05^b^	0.03 ± 0.01^d^
Acetic acid	0.5 ± 0.02^c^	0.72 ± 0.09^b^	0.76 ± 0.03^b^	2.04 ± 0.09^a^
Malic acid	5.10 ± 0.17^a^	5.69 ± 0.78^a^	5.77 ± 0.75^a^	4.13 ± 0.88^a^
**Yield**				
Ethanol (g/g sugar consumed)	0.48 ± 0.06^a^	0.47 ± 0.02^ab^	0.46 ± 0.02^ab^	0.44 ± 0.01^b^
Acetic acid (g/g sugar consumed)	0.002 ± 0.0001^c^	0.003 ± 0.0004^b^	0.004 ± 0.0001^b^	0.013 ± 0.0004^a^
Glycerol (g/g sugar consumed)	0.033 ± 0.0008^c^	0.034 ± 0.0008^c^	0.040 ± 0.0012^b^	0.063 ± 0.0022^a^
Succinic acid (g/g sugar consumed)	0.009 ± 0.0002^a^	0.0008 ± 0.0003^c^	0.0053 ± 0.0003^b^	0.0002 ± 0.0001^d^
**Ethanol production (%v/v)**	12.81 ± 0.15^a^	12.68 ± 0.19^a^	12.10 ± 0.52^a^	8.71 ± 0.61^b^
**Sugar (g) required for 1% (v/v) ethanol**	16.36 ± 0.20^a^	16.53 ± 0.24^a^	17.04 ± 0.86^a^	17.57 ± 2.02^a^
**Fermentation time (h)**	220	205	235	320

Standard deviation was used to determine the variation around the mean of triplicate treatments with superscript letters denoting statistically significant differences (*P* < 0.05). SM200, control medium; MET, methionine as a sole nitrogen source; CYS, cysteine as sole nitrogen source; B5 Def, limiting pantothenic acid (10 µg/l).

Notable differences were observed in the production of acetic acid, succinic acid, and glycerol between treatments. The MET condition produced ∼50% higher levels of acetic acid yield paired with a 10-fold reduction in succinic acid yield relative to the SM200 control. Similarly, in the CYS condition, succinic acid yield was reduced by two-fold but with increases in both glycerol and acetic acid yields compared to SM200. B5 deficiency produced the most dramatic metabolic perturbations of all treatments. Glycerol yield doubled, acetic acid yield increased five-fold, and succinic acid production was almost entirely abolished (∼50-fold decrease relative to control). This indicated a clear perturbance in both carbon and redox metabolism under this condition, consistent with the known role of pantothenic acid as a CoA precursor and its involvement in the TCA cycle.

### Global transcriptomic analysis

Transcriptome profiling was performed to assess the different nitrogen sources and pantothenic acid deficiency impact on gene expression during the growth phase (5 g/l CO₂) and the stationary phase (45 g/l CO₂) of wine fermentation. A PCA analysis of the expression of 6239 *S. cerevisiae* genes revealed clear differentiation between two sampling points in each treatment. We observed a variability of 25.1% and 31.6% in the two dimensions at the 5 g/l CO_2_ sampling point (Fig. S1A) and 15.4% and 44.03% of the variability at the 45 g/l CO_2_ sampling point (Fig. S1B). All biological replicates clustered tightly, except for one outlier in the CYS condition, which was excluded to ensure accurate interpretation. Overall, the CYS treatment exhibited the greatest transcriptional variance at the early growth sampling point (5 g/l CO_2_), whereas the B5 Def treatment displayed the highest variance in the stationary phase sampling point (45 g/l CO_2_).

### Differential gene expression analysis

**Figure 2:**
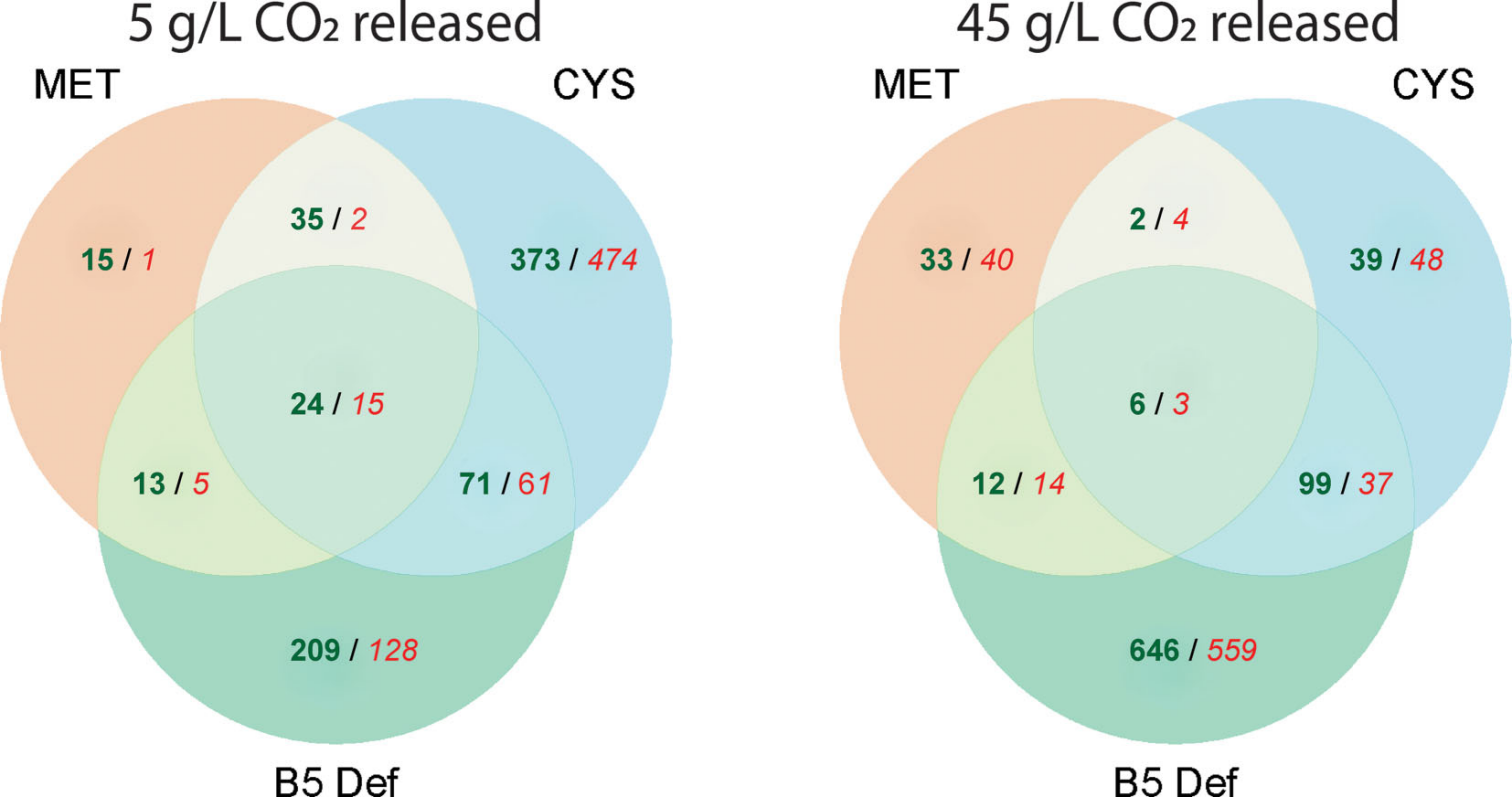
VENN diagram of differential gene expression for the fermentation with methionine (MET) and cysteine (CYS) as the sole nitrogen source compared to control. Number of genes that are upregulated (green) and downregulated (red/italic script) at 5 g/l CO2 released and upregulated (green) and downregulated (red italics) at 45 g/l CO2 released compared to the control condition (|log2FC| > 1, adjusted *P*-value < 0.05). VENN diagram created using VENNY v2.1 (Oliveros 2015). DEGs were identified for each treatment relative to the control (SM200) and depicted as volcano plots (Fig. S2). Across all conditions and fermentation stages, the most significantly up- and downregulated genes within each condition are easily visualized. We observed a moderate number of DEGs at both fermentation stages in the MET and CYS treatments, while the B5 Def treatment exhibited a notably larger transcriptional response, particularly during the stationary phase (45 g/l CO₂ released).

The differential expression analysis (Fig. 2) confirmed that pantothenic acid deficiency had the greatest impact on the transcriptome at 45 g/l (|log_2_FC| > 1, adjusted *P*-value < 0.05). Compared to the control condition, 317 genes were upregulated and 209 were downregulated at 5 g/l of released CO_2_, increasing to 763 upregulated and 613 downregulated genes at 45 g/l of released CO_2_, respectively. Cysteine as the sole nitrogen source also significantly impacted the gene expression profile mainly at 5 g/l CO_2_, with 503 genes upregulated and 552 downregulated and 146 and 92 at 45 g/l CO_2_, respectively. The methionine treatment produced only modest changes relative to the control. At 5 g/l of CO_2_, 87 genes were upregulated and 23 genes were downregulated, while 53 genes were upregulated and 61 genes downregulated at 45 g/l CO_2_. Overall, these results indicate that B5 Def triggered the most significant transcriptional response, followed by CYS, while MET exerted a smaller effect.

DEGs were identified for each treatment relative to the control (SM200) and depicted as volcano plots (Fig. S2). Across all conditions and fermentation stages, the most significantly up- and downregulated genes within each condition are easily visualized. We observed a moderate number of DEGs at both fermentation stages in the MET and CYS treatments, while the B5 Def treatment exhibited a notably larger transcriptional response, particularly during the stationary phase (45 g/l CO₂ released).

### Functional enrichment analysis

A Gene Ontology (GO) enrichment analysis was performed on both upregulated and downregulated gene lists. We observed a coordinated regulation of key metabolic processes including transport and transcription/translation. The GO enrichment results for the MET, CYS, and B5 Def conditions are presented in Fig. S3.1, S3.2, and S3.3, respectively.

### Effect of methionine on gene expression profile

#### Methionine induces amino acid transporter expression

During the growth phase, when methionine was provided as the sole nitrogen source, a large proportion of the induced genes belonged to GO categories related to transmembrane transport (Fig. S3.1). These included amino acid permeases *TNA1* and *AGP1* and oligopeptides transporters (*PTR2, OPT1, OPT2*). Notably, many of these, including *AGP1, PUT4, OPT1/2* and the highly upregulated general amino acid permease *GAP1* (log_2_FC = 2.98, Supplementary data S1) are under the control of nitrogen catabolite repression (NCR) (Schreve et al. 1998, Wiles et al. 2006). NCR acts to repress the uptake of less favourable nitrogen sources (e.g. methionine, proline, and urea) when preferred sources such as ammonium, glutamine, or asparagine are available (Cooper 1982, Godard et al. 2007). Interestingly, the differential expression of transporter genes between MET and SM200 (containing a mixture of ammonium and amino acids with high glutamine levels) conditions suggests a variation in NCR regulation, with repression being less effective when methionine is the sole nitrogen source.

Beyond general nitrogen transporter regulation, the expression of the methionine transporter *MUP1* remained largely unchanged between the above two conditions. This is surprising given that *MUP1* is a positive target of the Ssy1p regulatory system, which induces the permease expression in response to extracellular amino acids (Kosugi et al. 2001, Kodama et al. 2002). However, methionine remained available throughout the growth phase in SM200 fermentations likely explaining the absence of differential expression in *MUP1* expression. This aligns with the strong downregulation of *MUP1* observed under cysteine-only conditions (Supplementary data S3).

### Excess glutamate redirects nitrogen into arginine storage

**Figure 3:**
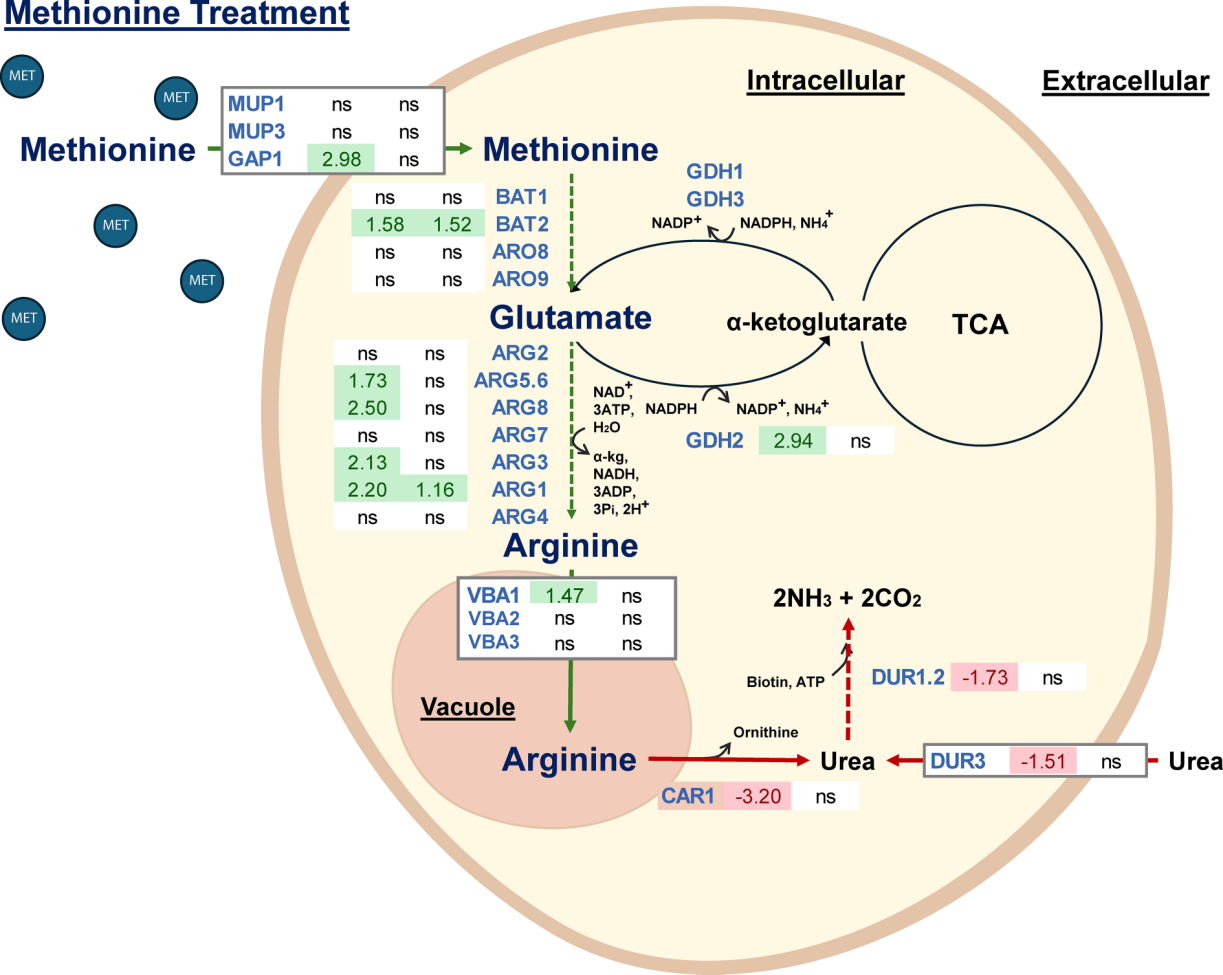
Differential expression (log₂FC) of genes involved in sulfur metabolism in *S. cerevisiae* LMD17 when methionine is the sole nitrogen source, sampled at 5 g/l cumulative CO₂ release (left box) and 45 g/l cumulative CO₂ release (right box). Gene names are overlaid on their corresponding enzymatic steps within the pathway. Significantly upregulated and downregulated genes are indicated in green (positive) and red (negative), respectively; genes not meeting the threshold (|log₂FC| ≥ 1, adjusted *P*-value < 0.05) are not significant (ns).

Under MET conditions (Fig. 3), the strong upregulation of *GDH2* could point to a substantial intracellular glutamate accumulation. This buildup originates directly from the assimilation of sulfur amino acids, which begins with transamination, generating glutamate from α-ketoglutarate. *GDH2* encodes glutamate dehydrogenase, which regenerates α-ketoglutarate from glutamate (Miller and Magasanik 1990). Such metabolic rerouting is consistent with the low efficiency of methionine in supporting yeast growth (Gutiérrez et al. 2013).

Consistent with this intracellular glutamate surplus, several genes for arginine biosynthesis (*ARG1, ARG5-6, ARG3, ARG8*) (Davis 1986) and the vacuolar arginine transporter VBA1 (Shimazu et al. 2005) were upregulated, while arginine catabolism genes (*CAR1, DUR1/2*) were strongly repressed (Fig. 3). Since glutamate is a direct precursor of arginine synthesis, these transcriptional changes suggest that surplus nitrogen was channelled into arginine synthesis prior to vacuolar storage. Arginine is a basic amino acid, known to be stored in the vacuole during early growth stages as a nitrogen reservoir, which can later be remobilized under nitrogen limitation (Crépin et al. 2014, Cools et al. 2020). Thus, during the MET treatment, excess nitrogen appears to be redirected into vacuolar arginine storage. Furthermore, to prevent the immediate breakdown of these reserves, genes involved in arginine catabolism are strongly repressed (Fig. 3).

### Methionine increases NADPH demand and acetate production

Methionine also influenced redox metabolism. Four aldehyde dehydrogenase genes (*ALD2-4, 6*) were significantly upregulated during the growth phase (Supplementary data S1), resulting in increasing acetate levels fourfold under MET fermentation compared to the control (Table 1). These genes are critical for maintaining redox balance and acetyl-CoA production (Flikweert et al. 1996, Saint-Prix et al. 2004, Duncan et al. 2025). The overexpression of ALD genes likely reflects elevated NADPH demand since cells utilizing methionine as the sole nitrogen source must synthesize all other amino acids *de novo*. This process necessitates a significantly higher demand for NADPH. Supporting this, the *MAE1* gene encoding the mitochondrial malic enzyme, another enzyme involved in maintaining NADP^+^/NADPH balance (Boles et al. 1998), was also upregulated.

### Thiamine biosynthesis is repressed under methionine conditions

MET conditions revealed a thiamine focused response on metabolism (Supplementary data S1, Fig. S2). Notably, a downregulation in genes from the subtelomeric family involved in thiamine precursor hydroxymethylpyrimidine (HMP) formation (*THI5,11–13)*, genes coding a trifunctional thiamine biosynthesis enzyme (*THI21,22*), a thiamine transporter (THI72), and enzymes induced in thiamine deficiency (*SNO2,3* and *SNZ2*,3) was observed (Llorente et al. 1999, Wightman and Meacock 2003, Ceschin et al. 2014).

Thiamine, in its active pyrophosphate form (TPP), plays essential roles in the cell. It acts as a cofactor for enzymes in central metabolic pathways like glycolysis, tricarboxylic acid cycle or pentose-phosphate pathway. Additionally, thiamine offers protection against cellular stress (Perli et al. 2020, Labuschagne and Divol 2021). In *S. cerevisiae*, thiamine can be obtained through uptake from the environment (the preferred and less energy-consuming route) or synthesized *de novo*. The activity of the thiamine biosynthesis pathway is tightly regulated by thiamine intracellular levels, with transcriptional repression occurring when levels are sufficient (Labuschagne and Divol 2021). Therefore, the downregulation of thiamine biosynthesis genes observed during methionine fermentation likely reflects lower metabolic activity and consequently, a reduced demand for thiamine compared to the control fermentation.

### Methionine metabolism supports methylthio-compounds formation

During the stationary phase, transcriptional differences were minimal (Supplementary data S2), with the exception of the upregulation of the *GCV1-3* genes when methionine was the sole nitrogen source. These genes encode the three subunits of the glycine cleavage complex, crucial for one-carbon metabolism and regeneration of 5-methyltetrahydrofolate (5-MTHF) from tetrahydrofolate (THF) (Piper et al. 2000). In yeast, 5-MTHF acts as a methyl group donor in various reactions, including the methylation of thiol groups (SH group) (Thomas and Surdin-Kerjan 1997). Interestingly, the formation of methylthio-containing molecules increased during the MET condition including 2ME, 3MTPA and 3MTPAc (Table S4). This upregulation of the GCV1-3 genes likely reflects the need to maintain sufficient levels of 5-MTHF as a methyl donor for the formation of these methylthio-containing compounds, necessitating its regeneration from THF (Jimenez-Lorenzo et al. 2021).

### Methionine catabolism relies on Ehrlich pathway activity

We previously reported that the Ehrlich pathway is the main metabolic route responsible for methionine catabolism in *S. cerevisiae* (Jimenez-Lorenzo et al. 2021). Each step of this route, widely explored for its contribution to the production of higher alcohol from amino and α-keto acids, relies on enzyme families (Hazelwood et al. 2008). Focusing on genes potentially involved in methionine catabolism and sulfur metabolism, differential expression was observed in only *BAT2* and *PDC6* during MET conditions (Supplementary data S1, S2).

The low number of DEGs is consistent with the nature of Ehrlich pathway enzymes, whose secondary catalytic activities remain governed by their primary metabolic functions. For example, alcohol dehydrogenases act primarily on acetaldehyde rather than methional, and their transcription is therefore unlikely to be controlled by methionine availability. Nevertheless, the early expression of the cytosolic branched-chain amino acid aminotransferase Bat2p, usually associated to the stationary phase (Colon et al. 2011), indicates that this enzyme contributed to methionine transamination from the beginning of fermentation. Likewise, we confirmed the increased expression of the pyruvate decarboxylase, Pdc6p, a minor isoform, which plays a role in the catabolism of amino acids (Dickinson et al. 2003). In contrast, no regulation of the SRS genes by methionine was detected.

### Effect of cysteine on gene expression profile

#### Cysteine triggers extensive remodelling of sulfur metabolism

**Figure 4:**
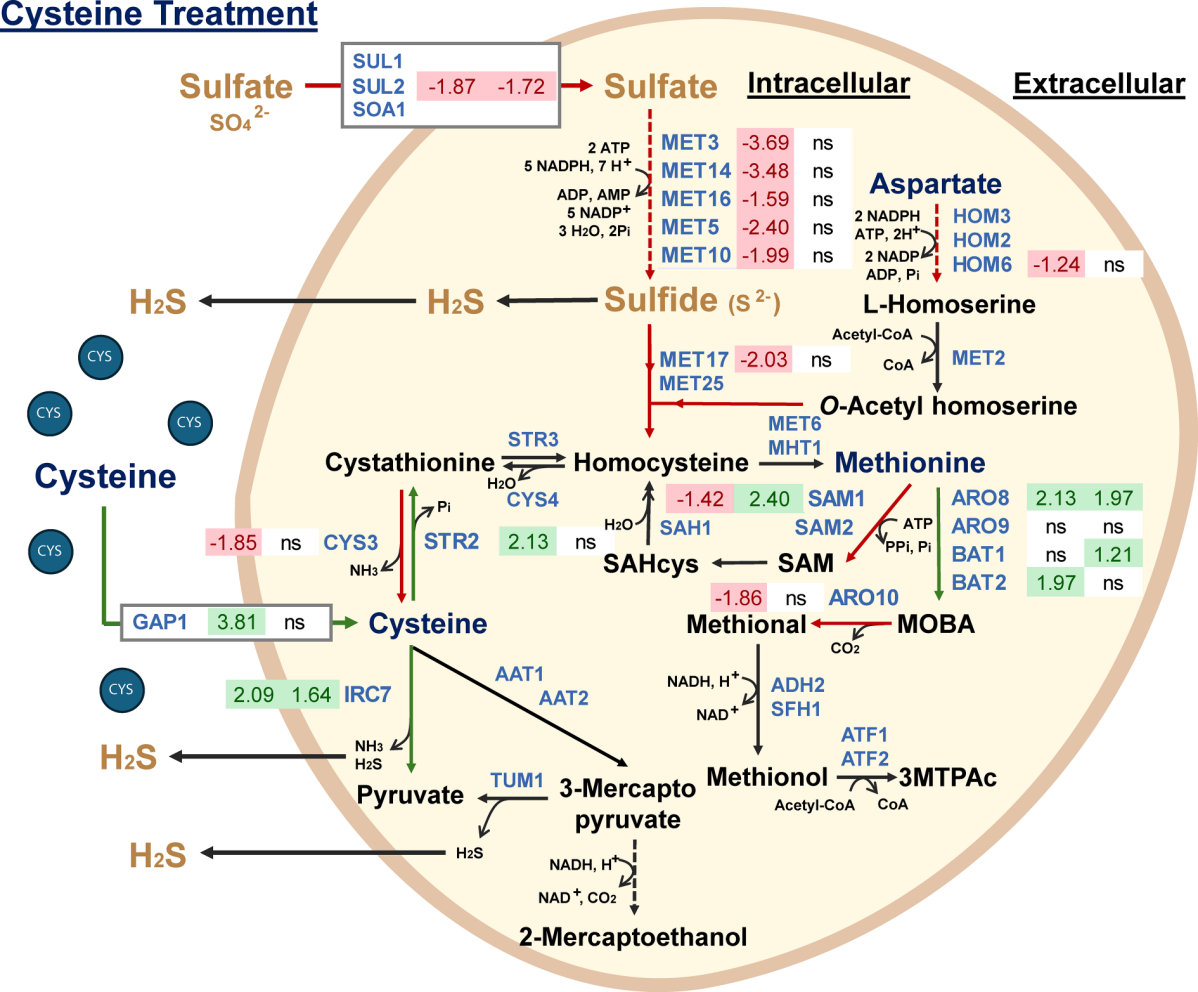
Differential expression (log₂FC) of genes involved in sulfur metabolism in *S. cerevisiae* LMD17 when cysteine is the sole nitrogen source, sampled at 5 g/l cumulative CO₂ release (left box) and 45 g/l cumulative CO₂ release (right box). Gene names are overlaid on their corresponding enzymatic steps within the pathway. Significantly upregulated and downregulated genes are indicated in green (positive) and red (negative), respectively; genes not meeting the threshold (|log₂FC| ≥ 1, adjusted *P*-value < 0.05) are not significant (ns).

While the impact of the MET treatment was limited to few genes in sulfur metabolism, a significant metabolic shift was triggered in the CYS treatment (Fig. 4). Indeed, genes for sulfate uptake and assimilation (*SUL2, MET3,5,10,14,16*) were downregulated. This trend included genes involved in the formation of O-acetyl homoserine and its further combination with sulfide to produce homocysteine (*HOM6, MET2,17/25*) as well as those involved in the formation of cysteine from methionine (*SAM1* and *CYS3*). These transcriptional changes were mainly observed during the growth phase (5 g/l of CO_2_ released).

In contrast to the repression of sulfur assimilation, we observed a substantial over expression of the genes responsible for the catabolism of cysteine, namely, *IRC7*, catalysing the cleavage of cysteine into pyruvate, ammonium and sulfide, *STR2* coding the cystathionine γ-synthase which irreversibly converts cysteine to cystathionine, and *ARO8* and *BAT1/2*, all involved in the transamination of amino acids in the first step of the Ehrlich pathway (Fig. 4). This reflects a shift in metabolic activity where the yeast solely uses cysteine as a source of sulfur, at the expense of the inorganic sulfur compounds provided in the medium. The cytotoxic effect of cysteine, previously described to affect cell growth (Kumar et al. 2006) may have contributed to this transcriptional response.

### Cysteine catabolism generates H_2_S and oxidative stress response

In *S. cerevisiae*, cysteine can be catabolized via two principal routes that both yield pyruvate and H_2_S. In the first, the transamination produces 3-mercaptopyruvate, which is further converted either to 2ME or to pyruvate and H_2_S (Kohl 2019). Through the second pathway, the desulfydrase Irc7p directly cleaves cysteine to pyruvate and H_2_S. This catabolism generates a sulfide surplus, as we have also observed in previous studies (Jimenez-Lorenzo et al. 2021).

The accumulation of H₂S is of particular physiological significance, as this compound is known to increase intracellular reactive oxygen species (ROS) in fungal species, including *Aspergillus niger* (Fu et al. 2014). A similar pattern in *S. cerevisiae* could account for the upregulation of genes linked to the cellular oxidant detoxification and ROS response (*CTA1, GTT1, CCP1, GRX1, CTT1, PRX1, GTO1)* during cysteine fermentation (Supplementary data S3, S4). Moreover, the upregulation of genes involved in downstream pyruvate metabolism (*MDH1,2, ALD2,3,4,6*) may be explained by a higher intracellular pyruvate content under CYS conditions, resulting from the increase in cysteine catabolism.

### Cysteine induces nitrogen starvation-like transcriptional response

When cysteine was used as the sole nitrogen source, we observed important changes in the gene expression profile compared to the SM200 control. This response was first governed by the low efficiency of cysteine to sustain *S. cerevisiae* growth and fermentation (Fig. 1). This observation was reflected by the under expression of genes associated with the translational initiation, purine nucleotide biosynthesis or cell wall organisation GO categories at 5 g/l of CO_2_ released (Fig. S3.2), when the metabolic activity under CYS conditions (fermentation rate: 0.3 g/l/h) was substantially lower than that of SM200 (1.0 g/l/h) (Table S3).

Another consequence of the poor growth was the activation of the carbon and nitrogen nutrient reserve systems (Supplementary data S3, Fig. S3.2). We observed the upregulation of several genes involved in glycogen biosynthesis process (*GDB1, PGM2, GLC3, GLG2, GSY2, IGD1, GAC1*), the transmembrane transport category (46 genes including genes involved in the transport of poor nitrogen sources including *DIP5, DUR3, PTR2, OPT2, DAL5, PUT4*), and in the catabolism of alternative nitrogen sources such as allantoin or proline (*DAL1, DAL3, DAL82, DAL2, DAL4, DAL7, PUT1, PUT2*). Interestingly, this pattern of regulation is similar to the transcriptional profile induced by the cells’ entry into stationary phase (Rossignol et al. 2003). Consequently, the nitrogen exhaustion occurring at this stage of fermentation induces a general response leading to the storage of carbohydrate as glycogen and trehalose (*GSY2, TPS1, NTH1*) during nitrogen limitation and stress (Hardwick et al. 1999, François and Parrou 2001). It appears that the low efficiency of cysteine assimilation by *S. cerevisiae* triggers a nitrogen starvation-like response in the transcriptome. In support of these observations, an upregulation of genes involved in autophagy (*ARC40, ARG82, ARG82, ATG1, ATG11, ATG14, ATG2, ATG3, ATG7, ATG8, AVT4, PCL5, PEP4, PRB1, SCH9, SEC17, YSP3*) was observed during the growth phase of CYS fermentation. Low metabolic activity causes a low availability of intracellular building blocks, such as amino acids or nucleic acids. To ensure yeast survival, this triggers autophagy to enable the turnover of these components. These cytoplasmic proteins and organelles are transported to the vacuole by autophagosomes, prior to further degradation by resident hydrolases (Scott et al. 1996). This process requires the presence of proteinase B (*PRB1*), with its upregulation observed (log2FC = 2.95, Supplementary data S3) in the CYS condition.

### Glutamate is redirected into arginine storage as thiamine biosynthesis is downregulated

As observed under MET conditions, CYS conditions also appeared to trigger the storage of arginine in the vacuole during the growth phase (5 g/l CO_2_). This is potentially a mechanism to fine-tune the management of intracellular glutamate pool. Genes involved in arginine production from glutamate (*ARG1, ARG4, ARG5,6*, Supplementary data S3) were also over expressed, however, in contrast to the MET treatment, no differential expression was found in genes associated with arginine catabolism (i.e. *CAR1, DUR1,2, DUR3*). This suggests that arginine accumulation is favoured under CYS conditions.

Beyond vacuolar arginine storage, excess glutamate appeared to be further redirected through the GABA pathway. An overexpression for all genes in the GABA pathway (*GAD1, UGA1, UGA2*) was observed in the CYS treatment (Supplementary data S3, Fig. S3). This is consistent with their role in glutamate catabolism (Bach et al. 2009), likely helping to mitigate the imbalance between the glutamate produced via cysteine transamination and its limited consumption for cellular anabolism.

Concurrent with these nitrogen-related responses, 8 of the 12 genes involved in thiamine biosynthesis (*THI5, THI11, THI13, THI21, SNZ2/3, THI12, THI22*) were downregulated when cysteine served as the sole nitrogen source (Supplementary data S3, Fig. S2, Fig. S3.2). This mirrors the response observed under MET conditions and reflects a common transcriptional response of the thiamine biosynthesis pathway to sulfur amino acids as sole nitrogen sources. Interestingly, an overexpression of this metabolic route has been previously reported during the growth phase compared to the stationary phase, and therefore directly related to the metabolic activity of yeast (Rossignol et al. 2003, Minebois et al. 2021). The coordinated downregulation of THI genes observed here under both sulfur amino acid conditions is therefore consistent with the slower fermentation performance of *S. cerevisiae* when relying solely on cysteine or methionine as nitrogen sources compared to the complete SM200 mediumbrk (Fig. 1).

### Tanscriptional response to pantothenic acid deficiency

#### Pantothenic acid deficiency impairs growth and fermentation

Pantothenic acid (vitamin B5) is a key precursor for the synthesis of CoA, acetyl-CoA, and acyl carrier proteins, involved in major metabolic reactions (Leonardi and Jackowski 2007). Notably, acetyl-CoA plays a central role in sulfur assimilation, serving as the acetyl donor for the formation of OAH, the organic backbone onto which sulfide is fixed to produce homocysteine (Jiranek et al. 1995). In *S. cerevisiae*, pantothenic acid can be imported via the pantothenate-proton symporter Fen2p (Stolz and Vielreicher 2003) when available in the medium or synthesized *de novo* from β-alanine and α-ketoisovalerate, the latter derived from valine catabolism (White et al. 2001, Perli et al. 2020). A deficiency in pantothenic acid impairs yeast growth and fermentation performance, increases acetate production modifies the profile of VSCs formation (Jimenez-Lorenzo et al. 2021).

### Limited transcriptional response in pantothenate biosynthesis pathway

Differential expression analysis revealed no significant upregulation of the specific genes responsible for pantothenic acid biosynthesis from carbon and nitrogen precursors (*ECM31, PAN5,6, FMS1*, Supplementary data S5) during the growth phase. This observation aligns with the constitutively low expression of *EMC31* and *PAN6*, previously described as unaffected by pantothenic acid availability during *S. cerevisiae* growth on 2% glucose minimal medium (Olzhausen et al. 2009). In contrast, genes involved in subsequent conversion of pantothenic acid to coenzyme A (*CAB1-5, CIS2, VHS3*) together with *PAN6* were upregulated under vitamin B5 deficiency at 45 g/l of CO_2_ release (Supplementary data S6). It is therefore possible that the transcriptional changes in the pantothenic biosynthetic pathway occur only after the complete depletion of this vitamin from the medium, at later fermentation stages.

### Sulfur metabolism is upregulated under pantothenic acid deficiency

**Figure 5:**
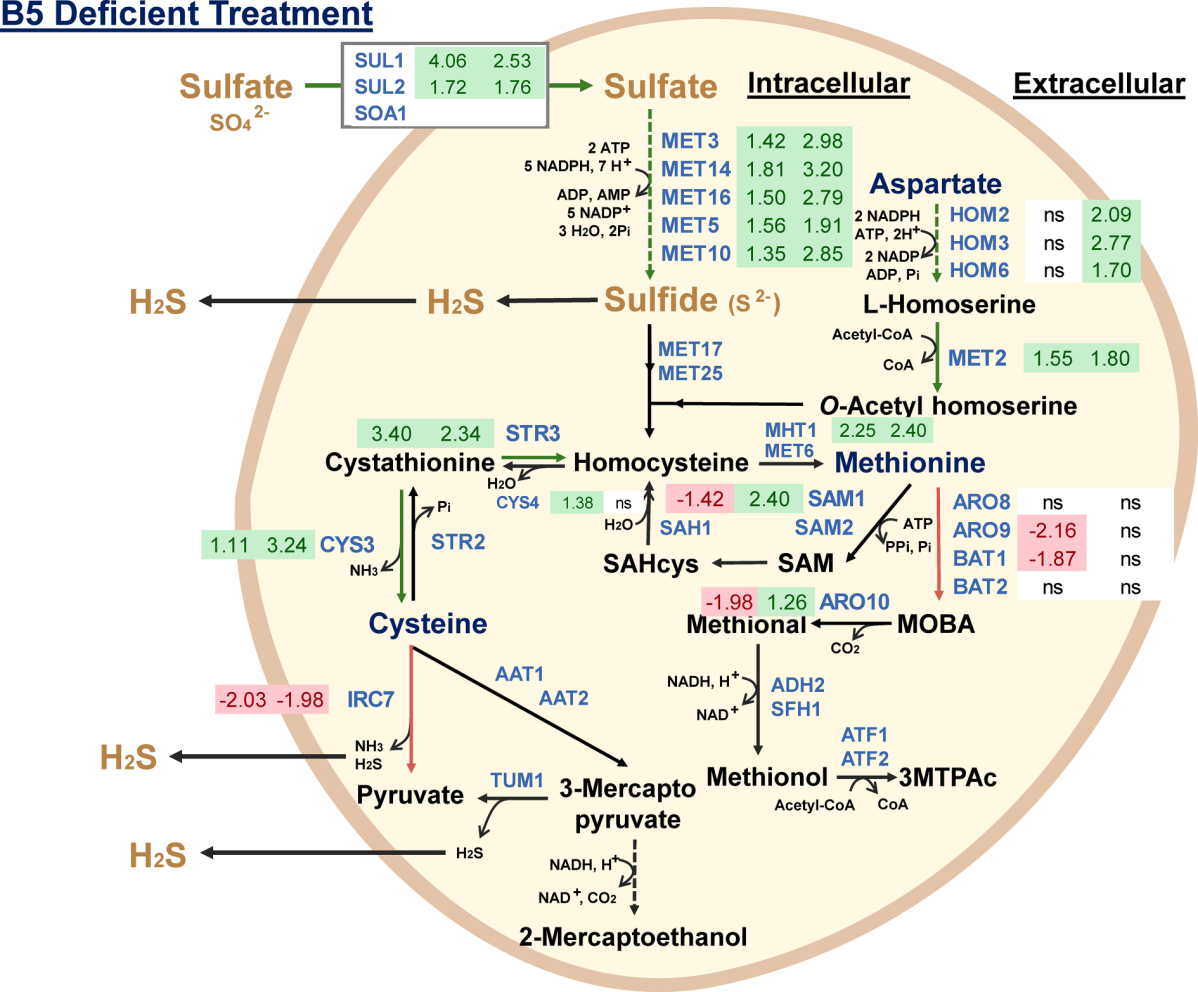
Differential expression (log₂FC) of genes involved in sulfur metabolism in *S. cerevisiae* LMD17 when vitamin B5 (pantothenic acid) is deficient, sampled at 5 g/l cumulative CO₂ release (left box) and 45 g/l cumulative CO₂ release (right box). Gene names are overlaid on their corresponding enzymatic steps within the pathway. Significantly upregulated and downregulated genes are indicated in green (positive) and red (negative), respectively; genes not meeting the threshold (|log₂FC| ≥ 1, adjusted *P*-value < 0.05) are not significant (ns).

At a global level, pantothenic acid deficiency elicited a transcriptional response focused on sulfur metabolism. The sulfate transporter *SUL1* and *SUL2*, all genes of the SRS pathway, most of the genes participating in the homocysteine, cysteine and methionine interconversions (*MHT1, CYS4, CYS3, STR3*) as well as *MET2*, which catalyses the acetyl-CoA-dependent conversion of homoserine to OAHS, were coordinately upregulated under pantothenic acid deficiency at both sampling points (Supplementary data S5; S6). Conversely, especially during the growth phase, genes involved in the catabolism of sulfur containing amino acids (*ARO8, BAT1, IRC7*) were downregulated (Fig. 5).

These transcriptional changes reflected a mobilization of sulfur resources towards amino acid biosynthesis, consistent with anabolic demands under stress. In agreement with this interpretation, pantothenic acid deficiency reduced the formation of VSCs compounds derived from methionine and cysteine catabolism (methionol, 3-methylthiopropionic acid, 3MTPAc, 2-mercaptoethanol, 3-mercaptopropanol) (Table S4). Interestingly, *ARO10*, involved in the catabolism of methionine through the Ehrlich pathway, was downregulated at 5 g/l of CO_2_ release but upregulated at 45 g/l of CO_2_. Moreover, the upregulation of the SRS pathway under pantothenic acid limitation may explain the higher production of H_2_S previously reported (Wang et al. 2003, Edwards and Bohlscheid 2007).

### Increased NADPH demand and downregulation of CoA-dependent pathways

The sulfur assimilation pathway is one of the most NADPH consuming metabolic processes, requiring four molecules of NADPH per molecule of sulfate assimilated (Thomas and Surdin-Kerjan 1997). During fermentation, the regeneration of NADP^+^ from NADPH is achieved by three major routes: the pentose-phosphate pathway (PPP), the mitochondrial malic enzyme (*MAE1*), and the activity of NADPH-dependent acetaldehyde dehydrogenase family (Celton et al. 2012). Under pantothenic acid deficiency, the upregulation of three PPP genes, including *GND2* (encoding an NADPH-dependent6-phosphogluconate dehydrogenase), likely reflects the need to counterbalance an increased demand for NADPH recycling to sustain the SRS pathway (Supplementary data S5, S6). Another important transcriptional response to pantothenic acid deficiency was the downregulation of genes associated with the tricarboxylic acid cycle and fatty acid biosynthesis (e.g. *FAA1* or *EEB1*) at both stages of fermentation (Supplementary data S5, S6, Figs S2, S3.3). This downregulation may result from the limited availability of CoA, as this cofactor (derived from pantothenic acid), is largely required in its acetyl-CoA form for these metabolic routes (Czumaj et al. 2020).

### Production of volatile sulfur compounds

**Figure 6:**
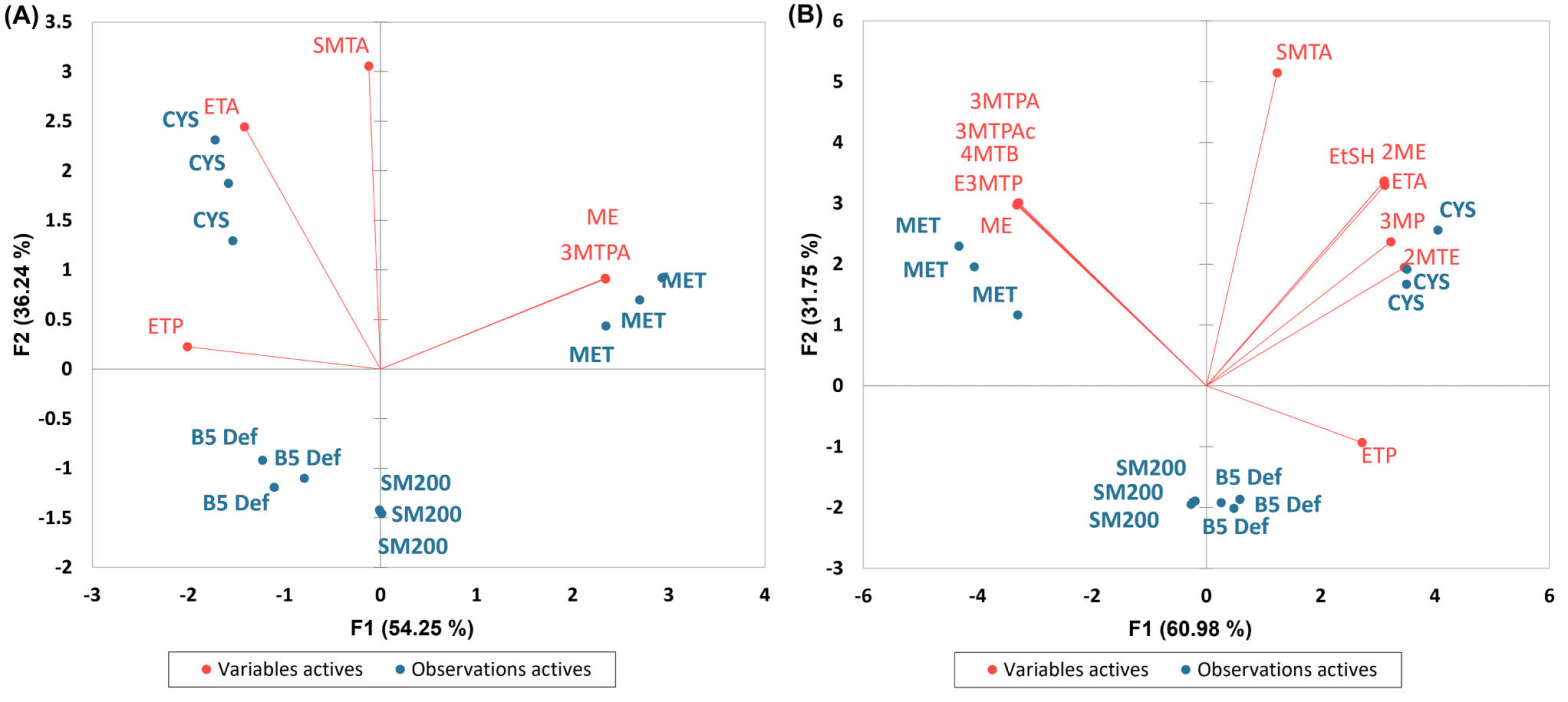
Principal component analysis (PCA) of volatile sulfur metabolites produced by *S. cerevisiae* LMD17 at 5 g/l CO₂ released (A) and 45 g/l CO₂ released (B) grown in a SM200 control medium; CYS, cysteine as sole nitrogen source; MET, methionine as a sole nitrogen source; B5 Def, limiting pantothenic acid medium. PCA A explained 90.50% and PCA B explained 92.73% of the total variance.

A PCA of the VSC profiles clearly separated the four conditions at both fermentation stages, explaining 90.50% and 92.73% of total variance at 5 g/l and 45 g/l CO₂ released, respectively (Fig. 6), confirming that VSC production was distinctly affected by each treatment. Across both sampling points, the MET and CYS treatments were the most divergent from one another, while the B5 deficiency and SM200 replicates clustered closely together, suggesting that pantothenic acid limitation had a comparatively modest effect on overall VSC profiles relative to the other treatments.

At the 5 g/l CO₂ released sampling point (Fig. 6A), the separation between conditions was primarily driven by the association of the CYS treatment with the thioesters SMTA and ETA, while the MET treatment was associated with ME and 3MTPA. At the 45 g/l CO₂ released sampling point (Fig. 6B), this divergence became more pronounced, with the MET treatment strongly associated with ME and the methylthio compounds 3MTPA, 3MTPAc, 4MTB, and E3MTP, while the CYS treatment was characterized by the thiols EtSH, 2ME, and 3MP, alongside the ethylthio compound ETP, the thioester ETA, and the methylthio compound 2MTE.

**Figure 7:**
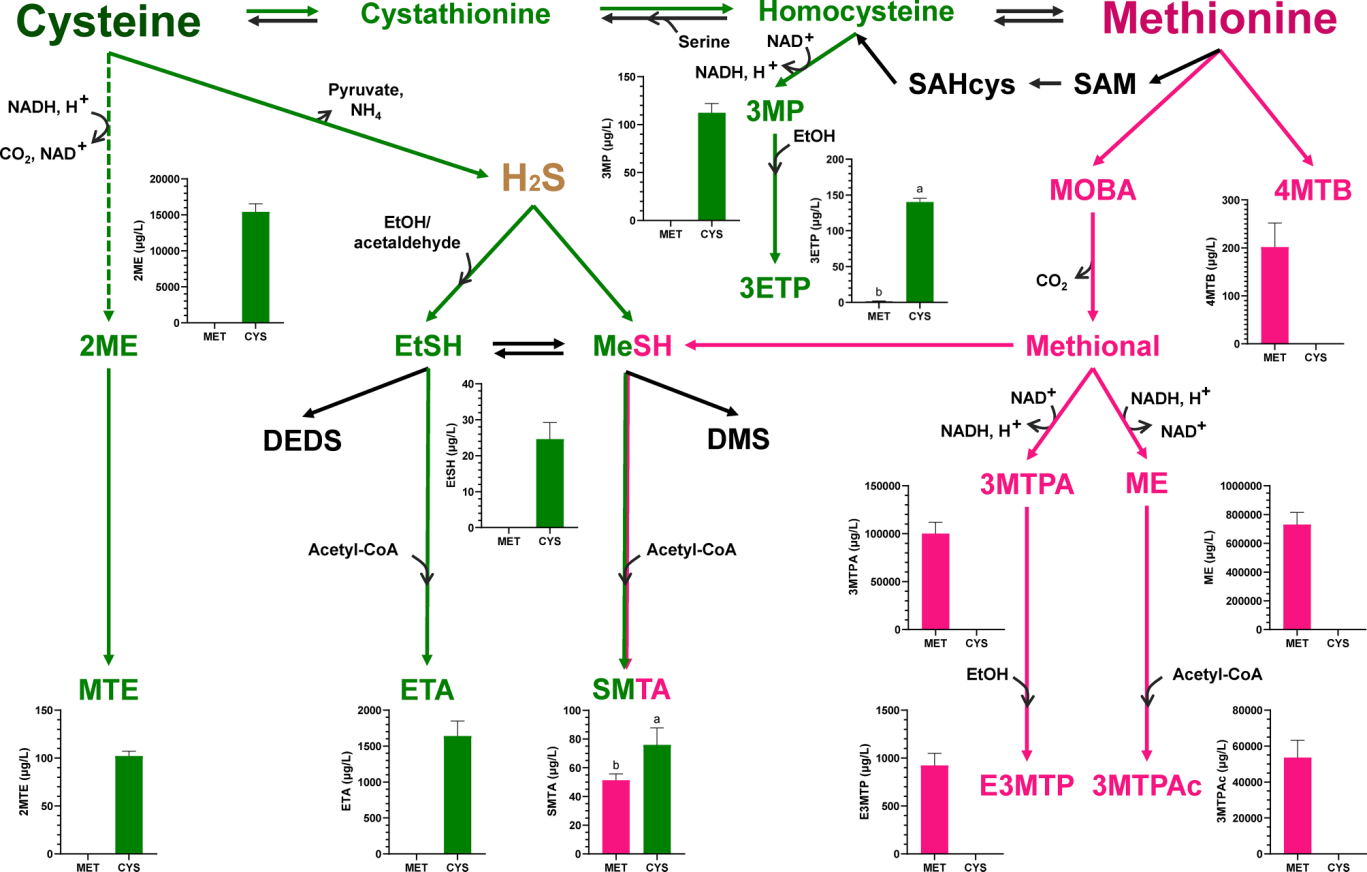
Volatile sulfur compound (VSC) production when cysteine (CYS) or methionine (MET) are the sole nitrogen source. Green arrows indicate flux from CYS metabolism and pink arrows indicate flux from MET metabolism. Standard deviation was used to determine the variation around the mean of triplicate treatments with superscript letters denoting statistically significant differences (*P* < 0.05). EtSH, ethanethiol; DMS, dimethyl sulfide; SMTA, S methyl thioacetate; ETA, ethanethioic acid; DEDS, diethyl disulfide; 2ME, 2 mercaptoethanol; MTE, 2 (methylthio)ethanol; E3MTP, ethyl 3 (methylthio)propanoate; 3ETP, 3 ethylthio 1 propanol; 3MTPAc, 3 (methylthio)propyl acetate; 3MP, 3 mercaptopyruvate; ME, methanethiol; EtSH, ethanethiol; MeSH, methanethiol; 4MTB, 4 (methylthio)butan 1 ol; 3MTPA, 3 (methylthio)propanoic acid. VSC concentrations can be seen in table S4.

During the MET condition, metabolic flux was strongly directed towards the methionine-derived branch of sulfur metabolism (Fig. 7, pink arrows), as well as an increased flux through the Ehrlich-like pathway and associated expression of genes involved in the Ehrlich pathway. Methionol, 3-methylthiopropanoic acid (3MTPA), and 3MTPAc were ∼140-, 487-, and 4000-fold higher than in the control, respectively, while ethyl 3-(methylthio)propanoate (E3MTP), 4-methylthiobutanol (4MTB), and SMTA were exclusively detected or elevated under this condition. Notably, little to no flux was directed towards the cysteine branch, with 2ME, ethanethiol (EtSH), and 3MP all below detection limits, suggesting a strong partitioning of sulfur flux towards methionine catabolism at the expense of other sulfur metabolic routes. This metabolic partitioning is consistent with our transcriptional results, where the upregulation of Ehrlich pathway genes (*BAT2, PDC6, ALD2-4/6*) and carbon metabolism genes (*GCV1-3*) directly supports the enhanced flux towards methylthio-compound formation. As *IRC7* and *STR2* are downregulated, this could explain the lack of cysteine-derived VSCs.

In the CYS condition, metabolic flux was redirected through the cysteine degradation pathway, generating a broader and contrasting range of volatile sulfur byproducts. Production of 2ME and ethyl thioacetate (ETA) increased ∼92- and 3000-fold relative to the control, respectively, reflecting enhanced flux through cysteine catabolic pathways (Fig. 7, green arrows). Additionally, 3MP, EtSH, 2MTE, and 3-ethylthio-1-propanol (3ETP) were exclusively detected or elevated under CYS conditions. Conversely, compounds associated with methionine catabolism were markedly reduced, with ME approximately six-fold lower than the control, and 3MTPA and 3MTPAc reduced by approximately four-fold and falling below the detection limit, respectively, confirming a reorientation of sulfur flux away from VSCs deriving from methionine catabolism. SMTA was the most highly produced thioester under CYS conditions, approximately six-fold higher than in SM200, and alongside SMTA production highlights methanethiol (MeSH) as a central metabolic node connecting cysteine and methionine metabolism. SMTA was produced under both nitrogen source conditions via acetyl-CoA condensation with MeSH, though at notably higher levels under CYS conditions.

The VSC profiles observed under CYS conditions are strongly supported by the transcriptional data, as the expression of *IRC7* and *STR2* accounts for the elevated H₂S-derived compounds. Indeed, the upregulation of *ARO8* and *BAT1/2* supports the increased Ehrlich pathway-like flux from cysteine, and the downregulation of methionine biosynthesis genes (*MET17, HOM6, SAM1*) is reflected in the reduced methylthio compound production (Fig. 4).

The study reveals a distinct compartmentalized response in VSC formation, driven by the specific sulfur amino acid provided, whereby volatile compounds deriving directly from the catabolism of the supplied amino acid are produced in vast excess, becoming the overwhelmingly predominant features of the VSC profile. Notably, no significant changes were observed in the expression of key genes governing the interconversion between methionine and cysteine—specifically *MET6, MHT1, CYS4*, and *STR3*—despite the massive increase in their respective intracellular availability when supplied as the sole nitrogen source. This lack of transcriptional flexibility in this pathway provides a clear mechanistic basis for the highly partitioned VSC profiles observed between the two conditions.

The B5 deficiency condition showed the least differentiation from the control regarding overall VSC profiles, as reflected by its proximity to SM200 in the PCA (Fig. 6). Nevertheless, a consistent decrease was observed in the formation of VSCs derived via the Ehrlich pathway from methionine (ME, 3MTPA, 3MTPAc), homocysteine (3MP), and cysteine (2ME), all of which were reduced between 2- and 13-fold or fell below the detection limit relative to SM200, suggesting an overall reduction in sulfur amino acid catabolism through this pathway under pantothenic acid limitation. This is consistent with the known role of pantothenic acid as a CoA precursor, as reduced acetyl-CoA availability would be expected to limit both Ehrlich pathway activity and thioester formation. Conversely, 3ETP was ∼185-fold higher than in the control, with SMTA and 2MTE also elevated, suggesting a partial redirection of sulfur flux towards non-Ehrlich catabolic routes under pantothenic acid deficiency (Table S4).

At the transcriptional level, the downregulation of Ehrlich pathway and cysteine catabolic genes (*ARO9, BAT1, IRC7*) directly accounts for the reduction in amino acid-derived VSCs, while the upregulation of the SRS pathway provides a mechanistic basis for the elevated production of H₂S-derived compounds such as 3ETP and SMTA through alternative catabolic routes.

## Conclusion

This study demonstrates that sulfur amino acid availability and pantothenic acid deficiency induce distinct transcriptional and metabolic states in *S. cerevisiae*, resulting in divergent patterns of VSC formation. These nutritional conditions impact the yeast through specific mechanisms. Pantothenic acid (B5) deficiency exerted the most significant impact, resulting in impaired fermentation performance and altered sulfur assimilation. It notably reduced the production of Ehrlich-derived VSCs while increasing non-Ehrlich catabolic by-products. While serving as a poor nitrogen source, methionine’s influence as sole nitrogen source remained moderate. It primarily remodelled the transcriptome to alter amino acid transporter expression, stimulate arginine biosynthesis and vacuolar storage, and increase NADPH demand. Met-driven flux was specifically directed toward methylthio-VSCs via the Ehrlich pathway. In contrast, cysteine triggered a nitrogen starvation-like response, characterized by the repression of ribosome biogenesis and the activation of nutrient storage pathways. This shifted metabolic flux toward cysteine catabolism, producing a broader and more diverse range of volatiles compared to methionine. Ultimately, methanethiol was identified as the central metabolic node, acting as the critical intermediate that links these distinct cysteine and methionine catabolic routes.

These findings open significant perspectives for both fundamental biology and the wine industry. From a fundamental standpoint, they raise questions about the relative contribution of transcriptional regulation, enzymatic crowding and metabolic control in maintaining this metabolic partitioning. For winemaking, these results offer concrete levers to modulate wine aromatic profiles. By fine-tuning the methionine and cysteine availability or vitamin B5 levels in musts through yeast-based nutrient supplementation, producers could control VSC production, effectively avoiding reductive off-flavors while optimizing overall sensory quality.

## Supplementary Material

foag022_Supplemental_Files

## References

[bib1] Allen T. The electrical sensing zone method of particle size distribution determination (the Coulter principle). In Particle Size Measurement. Dordrecht: Springer Netherlands, 1990, 455–82. 10.1007/978-94-009-0417-0

[bib2] Arfi K, Spinnler H, Tache R et al. Production of volatile compounds by cheese-ripening yeasts: requirement for a methanethiol donor for S-methyl thioacetate synthesis by *Kluyveromyces lactis*. Appl Microbiol Biotechnol. 2002;58:503–10. 11954798 10.1007/s00253-001-0925-0

[bib3] Bach B, Sauvage F, Dequin S et al. Role of γ-aminobutyric acid as a source of nitrogen and succinate in wine. Am J Enol Vitic. 2009;60:508–16. 10.5344/ajev.2009.60.4.508

[bib4] Blaiseau P, Isnard A, Surdin-Kerjan Y et al. Met31p and Met32p, two related zinc finger proteins, are involved in transcriptional regulation of yeast sulfur amino acid metabolism. Mol Cell Biol. 1997;17:3640–8. 10.1128/MCB.17.7.3640 9199298 PMC232216

[bib5] Blaiseau P, Thomas D. Multiple transcriptional activation complexes tether the yeast activator Met4 to DNA. EMBO J. 1998;17:6327–36. 10.1093/emboj/17.21.6327 9799240 PMC1170957

[bib6] Boles E, de Jong-Gubbels P, Pronk JT. Identification and characterization of MAE1, the *Saccharomyces cerevisiae* structural gene encoding mitochondrial malic enzyme. J Bacteriol. 1998;180:2875–82. 10.1128/JB.180.11.2875-2882.1998 9603875 PMC107252

[bib7] Celton M, Sanchez I, Goelzer A et al. A comparative transcriptomic, fluxomic and metabolomic analysis of the response of *Saccharomyces cerevisiae* to increases in NADPH oxidation. Bmc Genomics [Electronic Resource]. 2012;13:317. 10.1186/1471-2164-13-317 22805527 PMC3431268

[bib8] Ceschin J, Saint-Marc C, Laporte J et al. Identification of yeast and human 5-aminoimidazole-4-carboxamide-1-β-d-ribofuranoside (AICAr) transporters. J Biol Chem. 2014;289:16844–54. 10.1074/jbc.M114.551192 24778186 PMC4059127

[bib9] Chomczynski P, Sacchi N. Single-step method of RNA isolation by acid guanidinium thiocyanate-phenol-chloroform extraction. Anal Biochem. 1987;162:156–9. 10.1016/0003-2697(87)90021-2 2440339

[bib10] Colon M, Hernandez F, Lopez K et al. *Saccharomyces cerevisiae* Bat1 and Bat2 aminotransferases have functionally diverged from the ancestral-like *Kluyveromyces lactis* orthologous enzyme. PLoS One. 2011;6:e16099. 10.1371/journal.pone.0016099 21267457 PMC3022659

[bib11] Cools M, Lissoir S, Bodo E et al. Nitrogen coordinated import and export of arginine across the yeast vacuolar membrane. PLoS Genet. 2020;16:e1008966. 10.1371/journal.pgen.1008966 32776922 PMC7440668

[bib12] Cooper TG. Nitrogen metabolism in *Saccharomyces cerevisiae*. In: The Molecular Biology of the Yeast Saccharomyces: Metabolism and Gene Expression, Vol. 2, Cold Spring Harbor, NY: Cold Spring Harbor Laboratory, 1982, 39–99.

[bib13] Crépin L, Sanchez I, Nidelet T et al. Efficient ammonium uptake and mobilization of vacuolar arginine by *Saccharomyces cerevisiae* wine strains during wine fermentation. Microb Cell Fact. 2014;13:1–13. 25134990 10.1186/s12934-014-0109-0 PMC4244049

[bib14] Czumaj A, Szrok-Jurga S, Hebanowska A et al. The pathophysiological role of CoA. Int J Mol Sci. 2020;21:9057. 10.3390/ijms21239057 33260564 PMC7731229

[bib15] Davis RH. Compartmental and regulatory mechanisms in the arginine pathways of *Neurospora crassa* and *Saccharomyces cerevisiae*. Microbiol Rev. 1986;50:280–313. 10.1128/mr.50.3.280-313.1986 2945985 PMC373072

[bib16] Dickinson JR, Salgado LEJ, Hewlins MJ. The catabolism of amino acids to long chain and complex alcohols in *Saccharomyces cerevisiae*. J Biol Chem. 2003;278:8028–34. 10.1074/jbc.M211914200 12499363

[bib17] Duncan JD, Setati ME, Divol B. The cellular symphony of redox cofactor management by yeasts in wine fermentation. Int J Food Microbiol. 2025;427:110966. 10.1016/j.ijfoodmicro.2024.110966 39536648

[bib18] Edwards CG, Bohlscheid JC. Impact of pantothenic acid addition on H2S production by *Saccharomyces* under fermentative conditions. Enzyme Microb Technol. 2007;41:1–4. 10.1016/j.enzmictec.2007.03.002

[bib19] Evers MS, Ramousse L, Morge C et al. To be or not to be required: yeast vitaminic requirements in winemaking. Food Microbiol. 2023;115:104330. 10.1016/j.fm.2023.104330 37567622

[bib20] Flikweert MT, van der Zanden L, Janssen WMTM et al. Pyruvate decarboxylase: an indispensable enzyme for growth of *Saccharomyces cerevisiae* on glucose. Yeast. 1996;12:247–57. 10.1002/(SICI)1097-0061(19960315)12:3<247::AID-YEA911>3.0.CO;2-I 8904337

[bib21] François J, Parrou JL. Reserve carbohydrates metabolism in the yeast *Saccharomyces cerevisiae*. FEMS Microbiol Rev. 2001;25:125–45. 11152943 10.1111/j.1574-6976.2001.tb00574.x

[bib22] Franco-Luesma E, Ferreira V. Quantitative analysis of free and bonded forms of volatile sulfur compouds in wine. basic methodologies and evidences showing the existence of reversible cation-complexed forms. J Chromatogr A. 2014;1359:8–15. 10.1016/j.chroma.2014.07.011 25064535

[bib23] Fu L, Hu K, Hu L et al. An antifungal role of hydrogen sulfide on the postharvest pathogens *Aspergillus niger* and *Penicillium italicum*. PLoS One. 2014;9:e104206. 10.1371/journal.pone.0104206 25101960 PMC4125178

[bib24] Godard P, Urrestarazu A, Vissers S et al. Effect of 21 different nitrogen sources on global gene expression in the yeast *Saccharomyces cerevisiae*. Mol Cell Biol. 2007;27:3065–86. 10.1128/MCB.01084-06 17308034 PMC1899933

[bib25] Goedhart J, Luijsterburg MS. VolcaNoseR is a web app for creating, exploring, labeling and sharing volcano plots. Sci Rep. 2020;10:20560. 10.1038/s41598-020-76603-3 33239692 PMC7689420

[bib26] Gutiérrez A, Beltran G, Warringer J et al. Genetic basis of variations in nitrogen source utilization in four wine commercial yeast strains. PLoS One. 2013;8:e67166. 23826223 10.1371/journal.pone.0067166 PMC3691127

[bib27] Hansen J, Johannesen PF. Cysteine is essential for transcriptional regulation of the sulfur assimilation genes in *Saccharomyces cerevisiae*. Mol Gen Genet. 2000;263:535–42. 10.1007/s004380051199 10821189

[bib28] Hardwick JS, Kuruvilla FG, Tong JK et al. Rapamycin-modulated transcription defines the subset of nutrient-sensitive signaling pathways directly controlled by the tor proteins. Proc Natl Acad Sci USA. 1999;96:14866–70. 10.1073/pnas.96.26.14866 10611304 PMC24739

[bib29] Hazelwood LA, Daran J, Van Maris AJ et al. The Ehrlich pathway for fusel alcohol production: a century of research on *Saccharomyces cerevisiae* metabolism. Appl Environ Microb. 2008;74:2259–66. 10.1128/AEM.02625-07 PMC2293160 18281432

[bib30] Hopwood EM, Ahmed D, Aitken SM. A role for glutamate-333 of *Saccharomyces cerevisiae* cystathionine γ-lyase as a determinant of specificity. Biochim Biophys Acta, Proteins Proteom. 2014;1844:465–72. 10.1016/j.bbapap.2013.11.012 24291053

[bib31] Huang C, Walker ME, Fedrizzi B et al. The yeast TUM1 affects production of hydrogen sulfide from cysteine treatment during fermentation. FEMS Yeast Res. 2016;16:fow100. 10.1093/femsyr/fow100 27915245

[bib32] Jimenez-Lorenzo R, Bloem A, Farines V et al. How to modulate the formation of negative volatile sulfur compounds during wine fermentation?. FEMS Yeast Res. 2021;21:1–15. 10.1093/femsyr/foab038 PMC8310686 34191008

[bib33] Jiranek V, Langridge P, Henschke PA. Regulation of hydrogen sulfide liberation in wine-producing *Saccharomyces cerevisiae* strains by assimilable nitrogen. Appl Environ Microb. 1995;61:461–7. 10.1128/aem.61.2.461-467.1995 PMC167303 7574581

[bib34] Kodama Y, Omura F, Takahashi K et al. Genome-wide expression analysis of genes affected by amino acid sensor Ssy1p in Saccharomyces cerevisiae. Curr Genet. 2002;41:63–72. 10.1007/s00294-002-0291-1 12073087

[bib35] Kohl JB. A novel mouse model of sulfite oxidase deficiency: pathological changes in cysteine and H2S metabolism, Thesis Abstract, Universität zu Köln. 2019.

[bib36] Kosugi A, Koizumi Y, Yanagida F et al. MUP1, high affinity methionine permease, is involved in cysteine uptake by *Saccharomyces cerevisiae*. Biosci Biotechnol Biochem. 2001;65:728–31. 10.1271/bbb.65.728 11330701

[bib37] Kumar A, John L, Alam MM et al. Homocysteine-and cysteine-mediated growth defect is not associated with induction of oxidative stress response genes in yeast. Biochem J. 2006;396:61–69. 10.1042/BJ20051411 16433631 PMC1449999

[bib38] Kuras L, Cherest H, Surdin-Kerjan Y et al. A heteromeric complex containing the centromere binding factor 1 and two basic leucine zipper factors, Met4 and Met28, mediates the transcription activation of yeast sulfur metabolism. EMBO J. 1996;15:2519–29. 10.1002/j.1460-2075.1996.tb00609.x 8665859 PMC450184

[bib39] Labuschagne P, Divol B. Thiamine: a key nutrient for yeasts during wine alcoholic fermentation. Appl Microbiol Biotechnol. 2021;105:953–73. 10.1007/s00253-020-11080-2 33404836

[bib40] Landaud S, Helinck S, Bonnarme P. Formation of volatile sulfur compounds and metabolism of methionine and other sulfur compounds in fermented food. Appl Microbiol Biotechnol. 2008;77:1191–205. 10.1007/s00253-007-1288-y 18064452

[bib41] Lê S, Josse J, Husson F. FactoMineR: an R package for multivariate analysis. J Stat Softw. 2008;25:1–18.

[bib42] Leonardi R, Jackowski S. Biosynthesis of pantothenic acid and coenzyme A. EcoSal plus. 2007;2:10–1128. 10.1128/ecosalplus.3.6.3.4 PMC4950986 26443589

[bib43] Leonardi R, Zhang YM, Rock CO et al. Coenzyme A: back in action. Prog Lipid Res. 2005;44:125–53. 10.1016/j.plipres.2005.04.001 15893380

[bib44] Leonian LH, Lilly VG. The effect of vitamins on ten strains of *Saccharomyces cerevisiae*. American J Botany. 1942;29:459–64. 10.1002/j.1537-2197.1942.tb10235.x

[bib45] Llorente B, Fairhead C, Dujon B. Genetic redundancy and gene fusion in the genome of the baker’s yeast *Saccharomyces cerevisiae*: functional characterization of a three-member gene family involved in the thiamine biosynthetic pathway. Mol Microbiol. 1999;32:1140–52. 10.1046/j.1365-2958.1999.01412.x 10383756

[bib46] Miller SM, Magasanik B. Role of NAD-linked glutamate dehydrogenase in nitrogen metabolism in *Saccharomyces cerevisiae*. J Bacteriol. 1990;172:4927–35. 10.1128/jb.172.9.4927-4935.1990 1975578 PMC213147

[bib47] Minebois R, Lairón-Peris M, Barrio E et al. Metabolic differences between a wild and a wine strain of *Saccharomyces cerevisiae* during fermentation unveiled by multi-omic analysis. Environ Microbiol. 2021;23:3059–76. 10.1111/1462-2920.15523 33848053

[bib48] Moreira N, De Pinho PG, Santos C et al. Volatile sulphur compounds composition of monovarietal white wines. Food Chem. 2010;123:1198–203. 10.1016/j.foodchem.2010.05.086

[bib49] Natarajan K, Meyer MR, Jackson BM et al. Transcriptional profiling shows that Gcn4p is a master regulator of gene expression during amino acid starvation in yeast. Mol Cell Biol. 2001;21:4347–68. 10.1128/MCB.21.13.4347-4368.2001 11390663 PMC87095

[bib50] Oliveros JC. Venny. An interactive tool for comparing lists with Venn’s diagrams. 2015. Retrieved January 15, 2026, from https://bioinfogp.cnb.csic.es/tools/venny/index.html

[bib51] Olzhausen J, Schübbe S, Schüller H. Genetic analysis of coenzyme A biosynthesis in the yeast *Saccharomyces cerevisiae*: identification of a conditional mutation in the pantothenate kinase gene CAB1. Curr Genet. 2009;55:163–73. 10.1007/s00294-009-0234-1 19266201

[bib52] Perli T, Wronska AK, Ortiz-Merino RA et al. Vitamin requirements and biosynthesis in *Saccharomyces cerevisiae*. Yeast. 2020;37:283–304. 10.1002/yea.3461 31972058 PMC7187267

[bib53] Perpete P, Duthoit O, De Maeyer S et al. Methionine catabolism in *Saccharomyces cerevisiae*. FEMS Yeast Res. 2006;6:48–56. 10.1111/j.1567-1356.2005.00005.x 16423070

[bib54] Piper MD, Hong S, Ball GE et al. Regulation of the balance of one-carbon metabolism in *Saccharomyces cerevisiae*. J Biol Chem. 2000;275:30987–95. 10.1074/jbc.M004248200 10871621

[bib55] Rollero S, Bloem A, Camarasa C et al. Combined effects of nutrients and temperature on the production of fermentative aromas by Saccharomyces cerevisiae during wine fermentation. Appl Microbiol Biotechnol. 2015;99:2291–304. 10.1007/s00253-014-6210-9 25412578

[bib56] Rossignol T, Dulau L, Julien A et al. Genome-wide monitoring of wine yeast gene expression during alcoholic fermentation. Yeast. 2003;20:1369–85. 10.1002/yea.1046 14663829

[bib57] Sablayrolles J, Barre P, Grenier P. Design of a laboratory automatic system for studying alcoholic fermentations in anisothermal enological conditions. Biotechnol Tech. 1987;1:181–4. 10.1007/BF00227557

[bib58] Saint-Prix F, Bönquist L, Dequin S. Functional analysis of the ALD gene family of *Saccharomyces cerevisiae* during anaerobic growth on glucose: the NADP -dependent Ald6p and Ald5p isoforms play a major role in acetate formation. Microbiology. 2004;150:2209–20. 10.1099/mic.0.26999-0 15256563

[bib59] Santiago M, Gardner RC. The IRC7 gene encodes cysteine desulphydrase activity and confers on yeast the ability to grow on cysteine as a nitrogen source. Yeast. 2015;32:519–32. 10.1002/yea.3076 25871637

[bib60] Schreve JL, Sin JK, Garrett JM. The *Saccharomyces cerevisiae* YCC5 (YCL025c) gene encodes an amino acid permease, Agp1, which transports asparagine and glutamine. J Bacteriol. 1998;180:2556–9. 10.1128/JB.180.9.2556-2559.1998 9573211 PMC107201

[bib61] Scott SV, Hefner-Gravink A, Morano KA et al. Cytoplasm-to-vacuole targeting and autophagy employ the same machinery to deliver proteins to the yeast vacuole. Proc Natl Acad Sci USA. 1996;93:12304–8. 10.1073/pnas.93.22.12304 8901576 PMC37986

[bib62] Shimazu M, Sekito T, Akiyama K et al. A family of basic amino acid transporters of the vacuolar membrane from *Saccharomyces cerevisiae*. J Biol Chem. 2005;280:4851–7. 10.1074/jbc.M412617200 15572352

[bib63] Singh S, Padovani D, Leslie RA et al. Relative contributions of cystathionine β-synthase and γ-cystathionase to H2S biogenesis via alternative trans-sulfuration reactions. J Biol Chem. 2009;284:22457–66. 10.1074/jbc.M109.010868 19531479 PMC2755967

[bib64] Smyth GK, Speed T. Normalization of cDNA microarray data. Methods. 2003;31:265–73. 10.1016/S1046-2023(03)00155-5 14597310

[bib65] Stolz J, Vielreicher M. Tpn1p, the plasma membrane vitamin B6 transporter of *Saccharomyces cerevisiae*. J Biol Chem. 2003;278:18990–6. 10.1074/jbc.M300949200 12649274

[bib66] Supek F, Bošnjak M, Škunca N et al. REVIGO summarizes and visualizes long lists of gene ontology terms. PLoS One. 2011;6:e21800. 10.1371/journal.pone.0021800 21789182 PMC3138752

[bib67] Tang D, Chen M, Huang X et al. SRplot: a free online platform for data visualization and graphing. PLoS One. 2023;18:e0294236. 10.1371/journal.pone.0294236 37943830 PMC10635526

[bib68] Thomas D, Jacquemin I, Surdin-Kerjan Y. MET4, a leucine zipper protein, and centromere-binding factor 1 are both required for transcriptional activation of sulfur metabolism in *Saccharomyces cerevisiae*. Mol Cell Biol. 1992;12:1719–27. 1549123 10.1128/mcb.12.4.1719-1727.1992 PMC369615

[bib69] Thomas D, Surdin-Kerjan Y. Metabolism of sulfur amino acids in *Saccharomyces cerevisiae*. Microbiol Mol Biol Rev. 1997;61:503–32. 9409150 10.1128/mmbr.61.4.503-532.1997 PMC232622

[bib70] Vermeulen C, Lejeune I, Tran T et al. Occurrence of polyfunctional thiols in fresh lager beers. J Agric Food Chem. 2006;54:5061–8. 10.1021/jf060669a 16819917

[bib71] Wang XD, Bohlscheid JC, Edwards CG. Fermentative activity and production of volatile compounds by *Saccharomyces* grown in synthetic grape juice media deficient in assimilable nitrogen and/or pantothenic acid. J Appl Microbiol. 2003;94:349–59. 10.1046/j.1365-2672.2003.01827.x 12588542

[bib72] White WH, Gunyuzlu PL, Toyn JH. *Saccharomyces cerevisiae* is capable of de novo pantothenic acid biosynthesis involving a novel pathway of β-alanine production from spermine. J Biol Chem. 2001;276:10794–800. 10.1074/jbc.M009804200 11154694

[bib73] Wightman R, Meacock PA. The THI5 gene family of *Saccharomyces cerevisiae*: distribution of homologues among the *Hemiascomycetes* and functional redundancy in the aerobicbiosynthesis of thiamin from pyridoxine. Microbiology. 2003;149:1447–60. 10.1099/mic.0.26194-0 12777485

[bib74] Wiles AM, Cai H, Naider F et al. Nutrient regulation of oligopeptide transport in *Saccharomyces cerevisiae*. Microbiology. 2006;152:3133–45. 10.1099/mic.0.29055-0 17005992

