# Supplementary material for: Cysteine, methionine, and pantothenic acid remodel the Saccharomyces cerevisiae transcriptome and volatile sulfur compound metabolome during alcoholic fermentation
Source: FEMS Yeast Res. 2026 Jul 7;26:foag022. doi: 10.1093/femsyr/foag022 (PMC13348253; doi:10.1093/femsyr/foag022)
Supplement: foag022_Supplemental_Files [file foag022_supplemental_files.zip › Figure and supplementary figure captions Jimenez et al 2026.docx]

**Main text figure/table captions:**

**Figure 1**. The cumulative weight loss primarily attributed to CO_2_ release during fermentation of synthetic grape must by *S. cerevisiae* yeast LMD17 in each treatment. Legend: SM200, control medium; CYS, cysteine as sole nitrogen source; MET, methionine as a sole nitrogen source; B5 Def, limiting pantothenic acid (10 µg/L). Samples were taken at 5 g/L and 45 g/L CO_2_ released (dashed lines) during fermentation. Each data point depicts the mean values of triplicates, while the error bars represent the standard deviation among the triplicate samples.

**Figure 2.** VENN diagram of differential gene expression for the fermentation with methionine (MET) and cysteine (CYS) as the sole nitrogen source compared to control. Number of genes that are upregulated (green) and downregulated (red) at 5 g/L CO_2_ released and upregulated (green) and downregulated (red italics) at 45 g/L CO_2_ released compared to the control condition (|log_2_FC| > 1, adjusted *p-*value < 0.05). VENN diagram created using VENNY v2.1 (Oliveros, 2015).

**Figure 3.** Differential expression (log₂FC) of genes involved in sulfur metabolism in *S. cerevisiae* LMD17 when methionine is the sole nitrogen source, sampled at 5 g/L cumulative CO₂ release (left box) and 45 g/L cumulative CO₂ release (right box). Gene names are overlaid on their corresponding enzymatic steps within the pathway. Significantly upregulated and downregulated genes are indicated in green and red, respectively; genes not meeting the threshold (|log₂FC| ≥ 1, adjusted *p*-value < 0.05) are not significant (ns).

**Figure 4.** Differential expression (log₂FC) of genes involved in sulfur metabolism in *S. cerevisiae* LMD17 when cysteine is the sole nitrogen source, sampled at 5 g/L cumulative CO₂ release (left box) and 45 g/L cumulative CO₂ release (right box). Gene names are overlaid on their corresponding enzymatic steps within the pathway. Significantly upregulated and downregulated genes are indicated in green and red, respectively; genes not meeting the threshold (|log₂FC| ≥ 1, adjusted *p*-value < 0.05) are not significant (ns).

**Figure 5.** Differential expression (log₂FC) of genes involved in sulfur metabolism in *S. cerevisiae* LMD17 when vitamin B5 (pantothenic acid) is deficient, sampled at 5 g/L cumulative CO₂ release (left box) and 45 g/L cumulative CO₂ release (right box). Gene names are overlaid on their corresponding enzymatic steps within the pathway. Significantly upregulated and downregulated genes are indicated in green and red, respectively; genes not meeting the threshold (|log₂FC| ≥ 1, adjusted *p*-value < 0.05) are not significant (ns).

**Figure 6.** Principal component analysis (PCA) of volatile sulfur metabolites produced by *S. cerevisiae* LMD17 at 5 g/L CO_2_ released (**A**) and 45 g/L CO_2_ released (**B**) grown in a SM200 control medium; CYS, cysteine as sole nitrogen source; MET, methionine as a sole nitrogen source; B5 Def, limiting pantothenic acid medium. PCA A explained 90.50% and PCA B explained 92.73% of the total variance.

**Figure 7**. Volatile sulfur compound (VSC) production when cysteine (CYS) or methionine (MET) are the sole nitrogen source. Green arrows indicate flux from CYS metabolism and pink arrows indicate flux from MET metabolism. Standard deviation was used to determine the variation around the mean of triplicate treatments with superscript letters denoting statistically significant differences (*p* < 0.05). EtSH, ethanethiol; DMS, dimethyl sulfide; SMTA, S‑methyl thioacetate; ETA, ethanethioic acid; DEDS, diethyl disulfide; 2ME, 2‑mercaptoethanol; MTE, 2‑(methylthio)ethanol; E3MTP, ethyl 3‑(methylthio)propanoate; 3ETP, 3‑ethylthio‑1‑propanol; 3MTPAc, 3‑(methylthio)propyl acetate; 3MP, 3‑mercaptopyruvate; ME, methanethiol; EtSH, ethanethiol; MeSH, methanethiol; 4MTB, 4‑(methylthio)butan‑1‑ol; 3MTPA, 3‑(methylthio)propanoic acid. VSC concentrations can be seen in table S4.

**Supplementary Material Captions:**

**Table S1**. The synthetic grape juice-like medium (SM) composition in the control medium.

**Table S2**. Trace elements, vitamins and amino acids, anaerobic factors and iron chloride stock used to prepare the grape juice-like synthetic medium.

**Table S3.** The fermentation parameters in each treatment.

***** Fermentation condition B5 Def did not reach 70 g/L sugar consumption; therefore 60 g/L was used instead. SM200, control medium; MET, methionine as a sole nitrogen source; CYS, cysteine as sole nitrogen source; B5 Def, limiting pantothenic acid (10 µg/L). Standard deviation was used to determine the variation around the mean of triplicate treatments with superscript letters denoting statistically significant differences (*p* < 0.05).

**Table S4.** The volatile sulfur compounds were quantified at the end of fermentation.

Standard deviation was used to determine the variation around the mean of triplicate treatments with superscript letters denoting statistically significant differences (*p* < 0.05). SM200, control medium; MET, methionine as a sole nitrogen source; CYS, cysteine as sole nitrogen source; B5 Def, limiting pantothenic acid (10 µg/L); EtSH, ethanethiol; DMS, dimethyl sulfide; DES, diethyl sulfide; SMTA, S‑methyl thioacetate; DMDS, dimethyl disulfide; ETA, ethylthio acetate; DEDS, diethyl disulfide; MAL, methional; 2ME, 2‑mercaptoethanol; MTHTP, 3‑(methylthio)propanal; 2MTE, 2‑(methylthio)ethanol; E3MTP, ethyl 3‑(methylthio)propanoate; 3MTPAc, 3‑(methylthio)propyl acetate; 3MP, 3‑mercaptopyruvate; ME, methanethiol; 3ETP, 3‑ethylthio‑1‑propanol; 4MTB, 4‑(methylthio)butan‑1‑ol; 3MTPA, 3‑(methylthio)propanoic acid; n.d., not detected.

**Figure S1**. Principal component analysis of the microarray data set for each replicate of *Saccharomyces cerevisiae* LMD17 at 5 g of CO_2_ released (A) and at 45 g of CO_2_ released (B) in each treatment: Control, SM200; Cys, cysteine as sole nitrogen source; Met, methionine as sole nitrogen source; DeP, deficient pantothenic acid. Cys_5g_2 did not cluster with the other replicates, and was removed from further analysis to ensure accuracy. The first two dimensions (Dim1 and Dim2) explain 25.07% and 31.61% of the variability (A) and 15.36% and 44.03% of the variability (B).

**Figure S2**. Volcano plots of differentially expressed genes (DEGs) in *S. cerevisiae* LMD17 under distinct nutritional conditions and fermentation stages. Panels depict methionine as the sole nitrogen source at 5 g/L cumulative CO₂ release (A) and 45 g/L cumulative CO₂ release (B); cysteine as the sole nitrogen source at 5 g/L cumulative CO₂ release (C) and 45 g/L cumulative CO₂ release (D); and pantothenic acid deficiency at 5 g/L cumulative CO₂ release (E) and 45 g/L cumulative CO₂ release (F). Each point represents one gene; significantly downregulated and upregulated DEGs are highlighted in green and purple, respectively (|log₂FC| ≥ 1, adjusted *p*-value < 0.05). Created with VolcaNoseR (Goedhart and Luijsterburg, 2020).

**Figure S3.1**. Gene Ontology (GO) term enrichment (adjusted *p*-value < 0.05) among downregulated (left) and upregulated (right) genes in *S. cerevisiae* LMD17 during fermentation with **methionine as the sole nitrogen source**, sampled at 5 g/L and 45 g/L cumulative CO₂ release. Each dot represents an enriched GO term, with dot size proportional to the number of genes annotated to that term and dot colour indicating the adjusted *p*-value. The Gene Ratio reflects the proportion of differentially expressed genes associated with each GO term relative to the total number of genes in that term. Generated with SR Plot (Tang et al., 2023).

**Figure S3.2**. Gene Ontology (GO) term enrichment (adjusted *p*-value < 0.05) among downregulated (left) and upregulated (right) genes in *S. cerevisiae* LMD17 during fermentation with **cysteine as the sole nitrogen source**, sampled at 5 g/L and 45 g/L cumulative CO₂ release. Each dot represents an enriched GO term, with dot size proportional to the number of genes annotated to that term and dot colour indicating the adjusted *p*-value. The Gene Ratio reflects the proportion of differentially expressed genes associated with each GO term relative to the total number of genes in that term. Generated with SR Plot (Tang et al., 2023).

**Figure S3.3**. Gene Ontology (GO) term enrichment (adjusted *p*-value < 0.05) among downregulated (left) and upregulated (right) genes in *S. cerevisiae* LMD17 during fermentation with **deficient levels of vitamin B5** (pantothenic acid), sampled at 5 g/L and 45 g/L cumulative CO₂ release. Each dot represents an enriched GO term, with dot size proportional to the number of genes annotated to that term and dot colour indicating the adjusted *p*-value. The Gene Ratio reflects the proportion of differentially expressed genes associated with each GO term relative to the total number of genes in that term. Generated with SR Plot (Tang et al., 2023).

**Supplementary data S1**: Differential gene expression results for SM200 vs MET at 5 g/L (DGE_SM200_vs_MET_5g.xlsx)

**Supplementary data S2:** Differential gene expression results for SM200 vs MET at 45 g/L (DGE_SM200_vs_MET_45g.xlsx)

**Supplementary data S3**: Differential gene expression results for SM200 vs CYS at 5 g/L (DGE_SM200_vs_CYS_5g.xlsx)

**Supplementary data S4:** Differential gene expression results for SM200 vs CYS at 45 g/L (DGE_SM200_vs_CYS_45g.xlsx)

**Supplementary data S5**: Differential gene expression results for SM200 vs B5Def at 5 g/L (DGE_SM200_vs_B5Def_5g.xlsx)

**Supplementary data S6**: Differential gene expression results for SM200 vs B5Def at 45 g/L (DGE_SM200_vs_B5DEF_45g.xlsx)
