# Supplementary figures and images for: Cysteine, methionine, and pantothenic acid remodel the Saccharomyces cerevisiae transcriptome and volatile sulfur compound metabolome during alcoholic fermentation
Source: FEMS Yeast Res. 2026 Jul 7;26:foag022. doi: 10.1093/femsyr/foag022 (PMC13348253; doi:10.1093/femsyr/foag022)

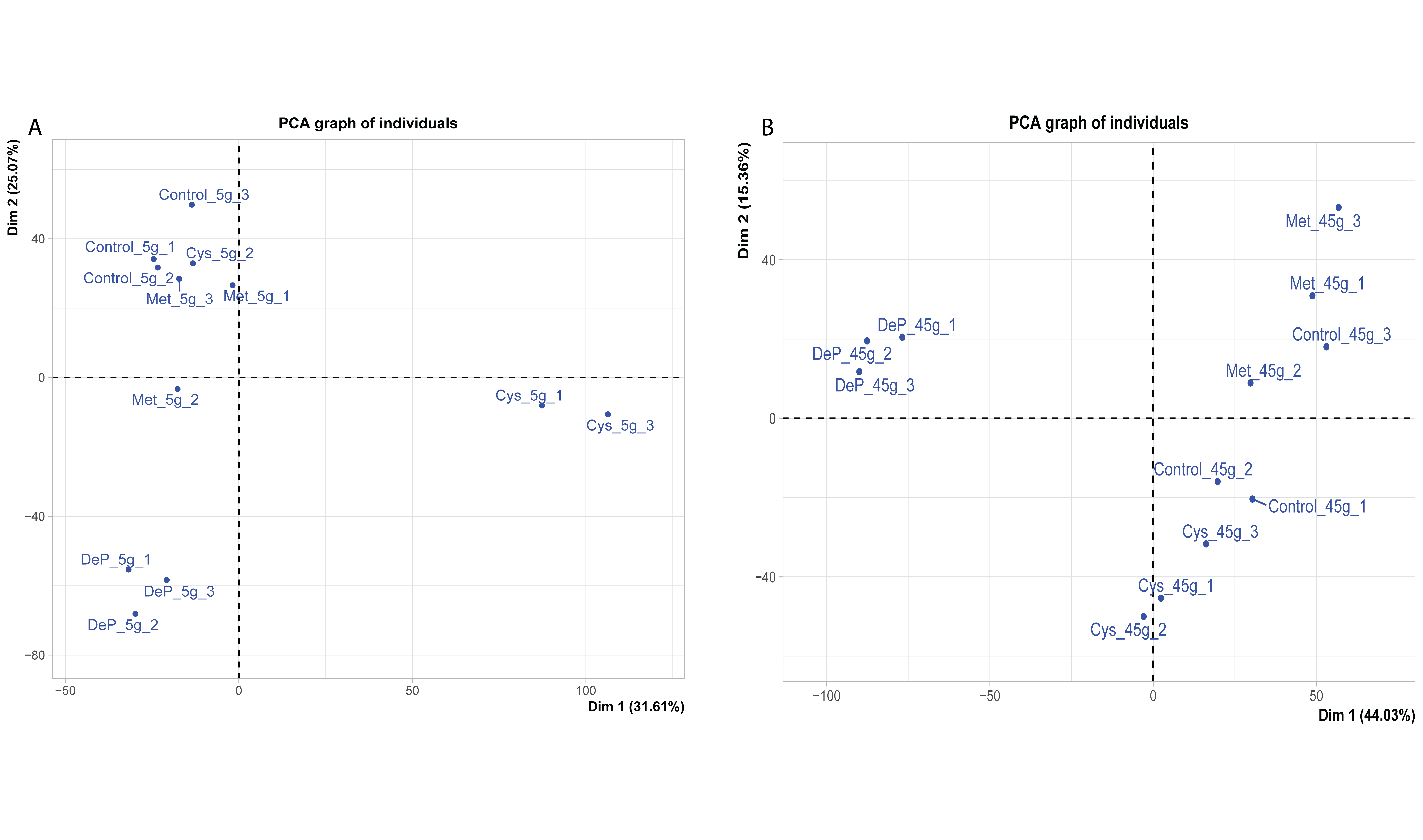

Supplement: foag022_Supplemental_Files [file foag022_supplemental_files.zip › Figure S1.tif]

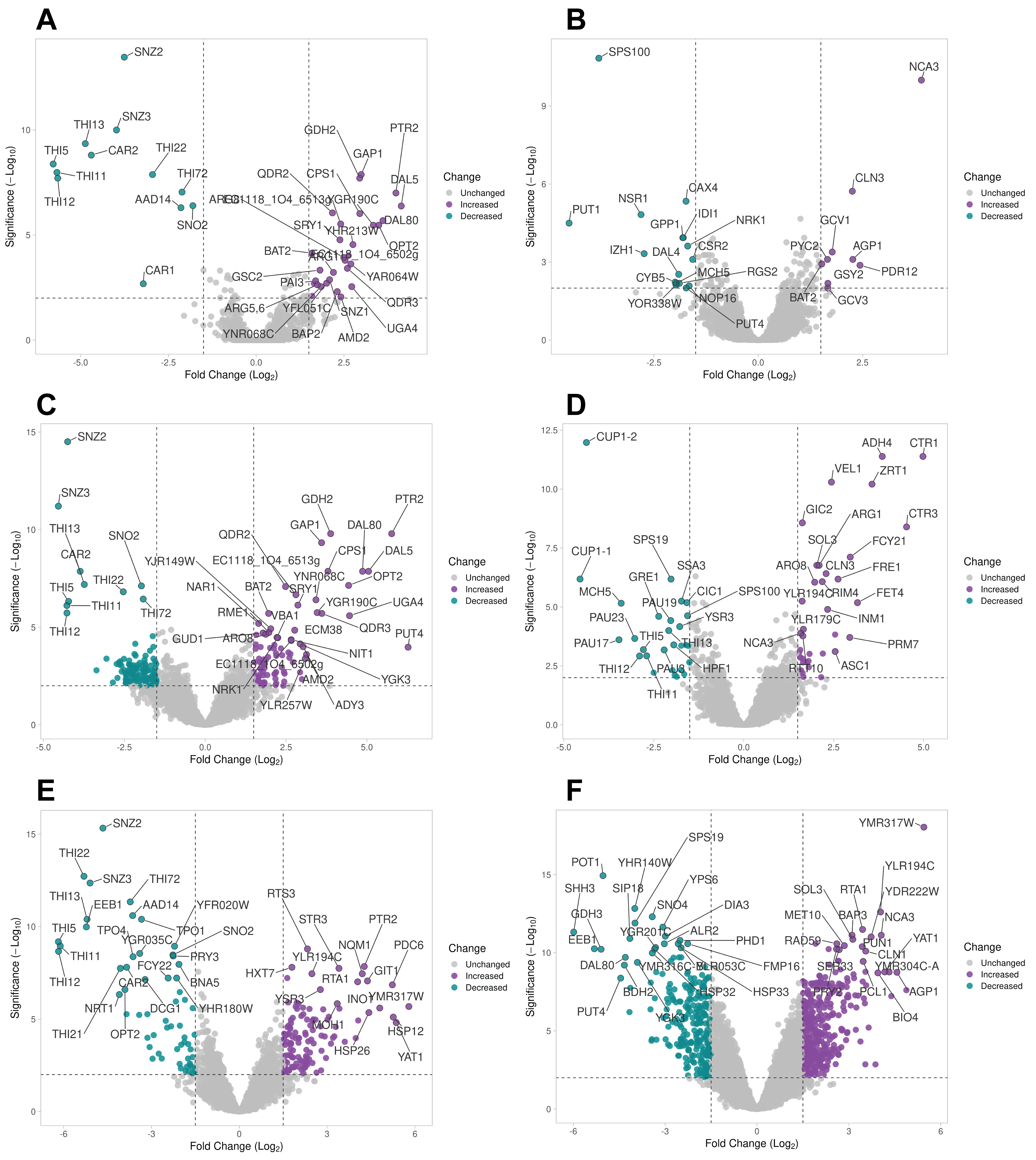

Supplement: foag022_Supplemental_Files [file foag022_supplemental_files.zip › Figure S2.tif]

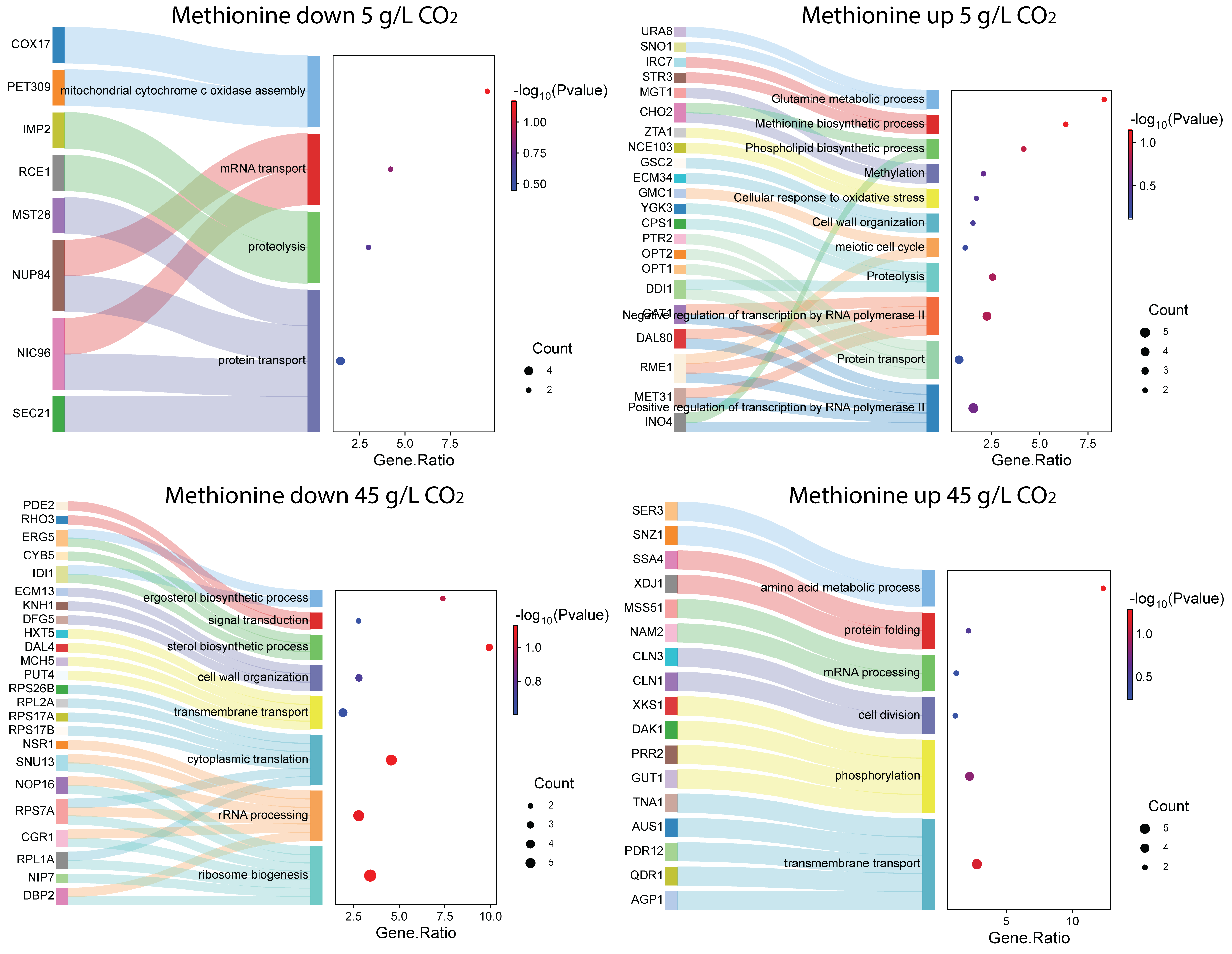

Supplement: foag022_Supplemental_Files [file foag022_supplemental_files.zip › Figure S3.1.tif]

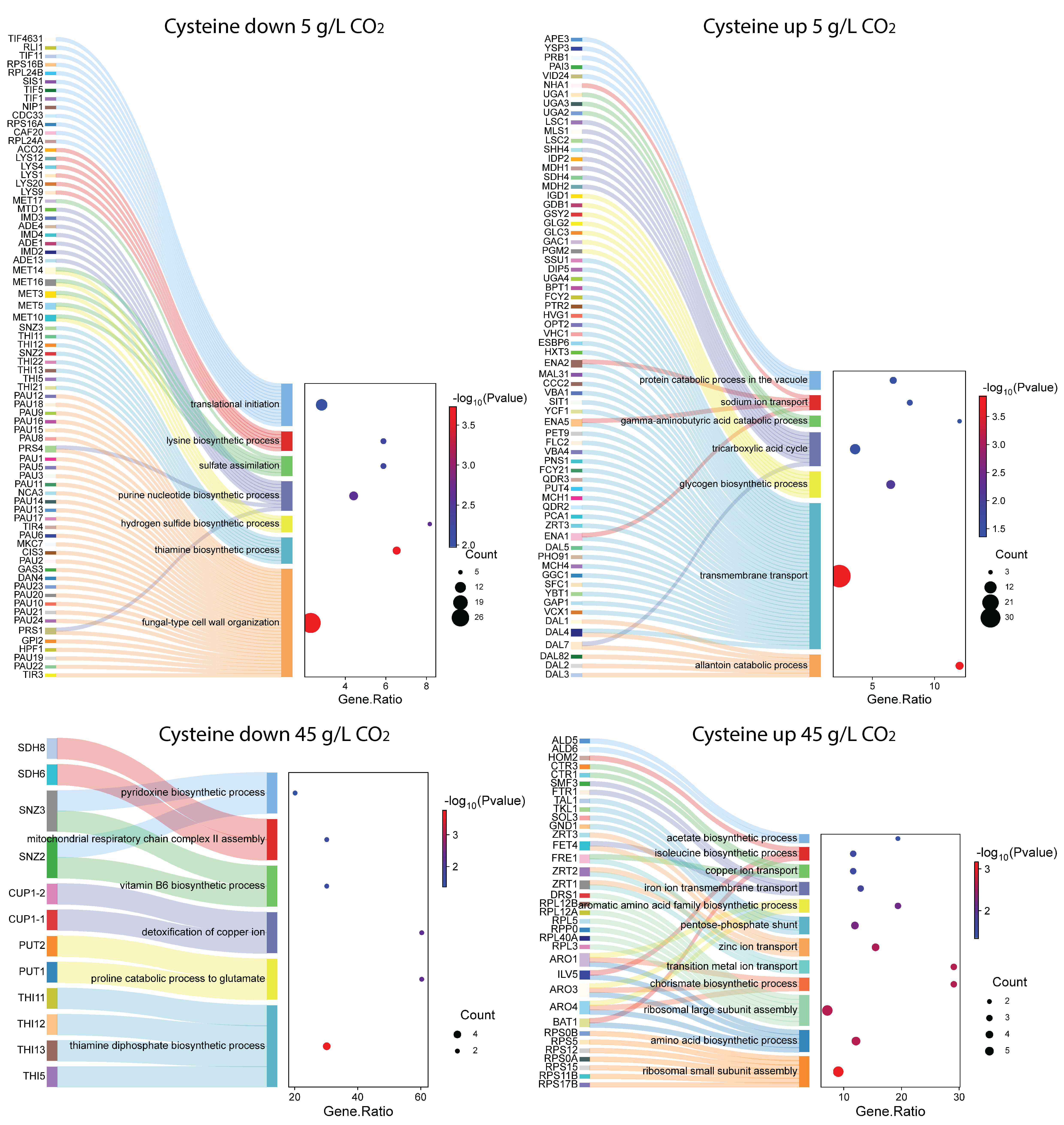

Supplement: foag022_Supplemental_Files [file foag022_supplemental_files.zip › Figure S3.2.tif]

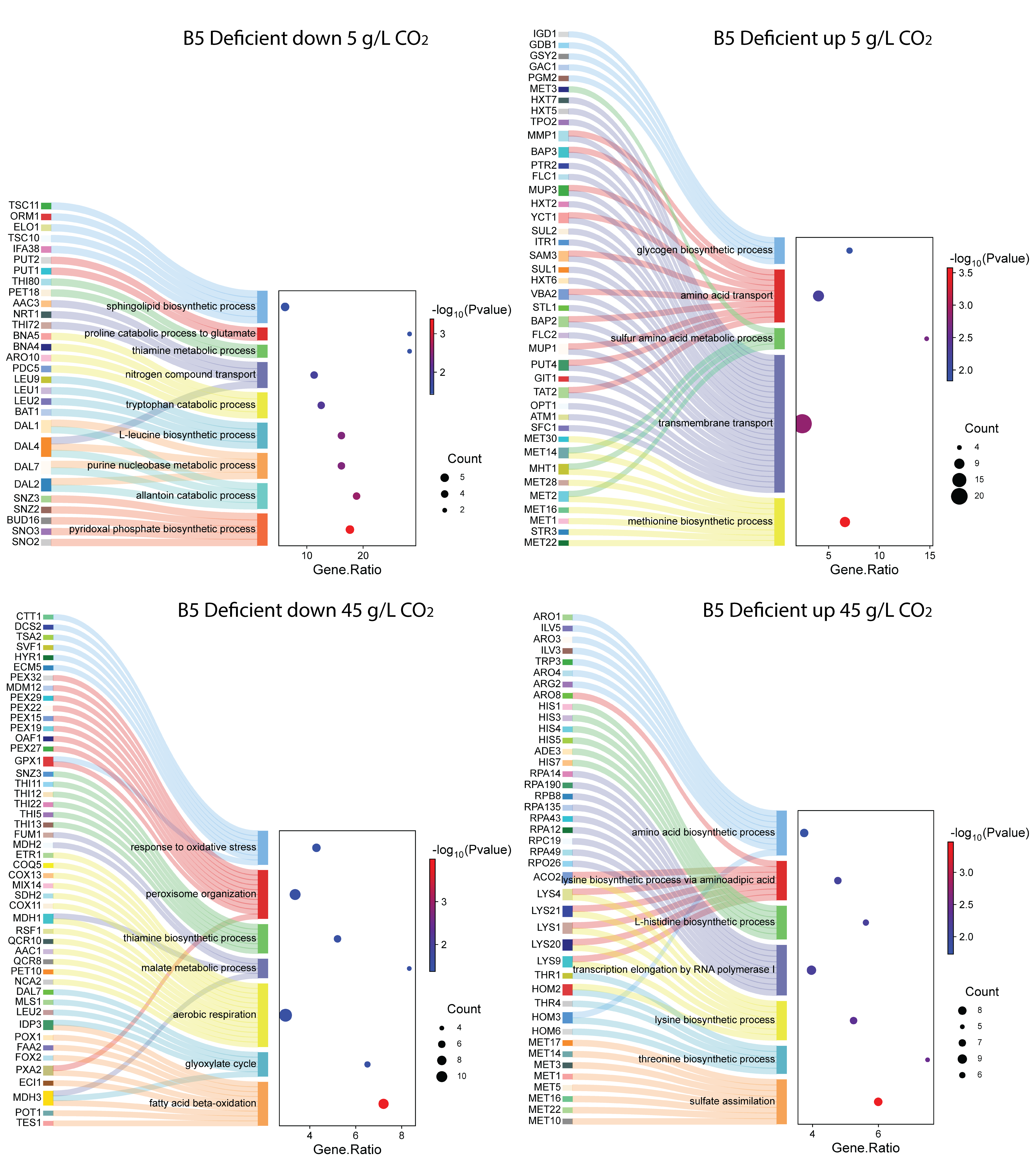

Supplement: foag022_Supplemental_Files [file foag022_supplemental_files.zip › Figure S3.3.tif]
